# Guidelines for the pharmacological treatment of COVID-19. The task-force/consensus guideline of the Brazilian Association of Intensive Care Medicine, the Brazilian Society of Infectious Diseases and the Brazilian Society of Pulmonology and Tisiology

**DOI:** 10.5935/0103-507X.20200039

**Published:** 2020

**Authors:** Maicon Falavigna, Verônica Colpani, Cinara Stein, Luciano Cesar Pontes Azevedo, Angela Maria Bagattini, Gabriela Vilela de Brito, José Miguel Chatkin, Sergio Cimerman, Mirian de Freitas Dal Ben Corradi, Clovis Arns da Cunha, Flávia Cordeiro de Medeiros, Haliton Alves de Oliveira Junior, Leandro Genehr Fritscher, Marcelo Basso Gazzana, Débora Dalmas Gräf, Lays Pires Marra, Jessica Yumi Matuoka, Michelle Silva Nunes, Daniela Vianna Pachito, Cássia Garcia Moraes Pagano, Patrícia do Carmo Silva Parreira, Rachel Riera, Amilton Silva Júnior, Bruno de Melo Tavares, Alexandre Prehn Zavascki, Regis Goulart Rosa, Felipe Dal-Pizzol

**Affiliations:** 1 Hospital Moinhos de Vento - Porto Alegre (RS), Brasil.; 2 Instituto para Avaliação de Tecnologia em Saúde, Universidade Federal do Rio Grande do Sul - Porto Alegre (RS), Brasil.; 3 Department of Health Research Methods, Evidence, and Impact, McMaster University - Hamilton, Canadá.; 4 Programa de Pós-Graduação em Ciências Médicas: Endocrinologia, Universidade Federal do Rio Grande do Sul - Porto Alegre (RS), Brasil.; 5 Hospital Sírio-Libanês - São Paulo (SP), Brasil.; 6 Disciplina de Emergências Clínicas, Universidade de São Paulo - São Paulo (SP), Brasil.; 7 Programa de Pós-Graduação em Medicina Tropical e Saúde Pública, Universidade Federal de Goiás - Goiânia (GO), Brasil.; 8 Centro Internacional de Pesquisa, Hospital Alemão Oswaldo Cruz - São Paulo (SP), Brasil.; 9 Pontifícia Universidade Católica do Rio Grande do Sul - Porto Alegre (RS), Brasil.; 10 Sociedade Brasileira de Pneumologia e Tisiologia - Brasília (DF), Brasil.; 11 Instituto de Infectologia Emílio Ribas - São Paulo (SP), Brasil.; 12 Sociedade Brasileira de Infectologia - São Paulo (SP), Brasil.; 13 Universidade Federal do Paraná - Curitiba (PR), Brasil.; 14 Programa de Pós-Graduação em Ciências Pneumológicas, Universidade Federal do Rio Grande do Sul - Porto Alegre (RS), Brasil.; 15 Serviço de Pneumologia e Cirurgia Torácica, Hospital Moinhos de Vento - Porto Alegre (RS), Brasil.; 16 Empresa Brasileira de Serviços Hospitalares - Brasília (DF), Brasil.; 17 Fundação Getúlio Vargas - São Paulo (SP), Brasil.; 18 Escola Paulista de Medicina, Universidade Federal de São Paulo - São Paulo (SP), Brasil.; 19 Hospital Alemão Oswaldo Cruz - São Paulo (SP), Brasil.; 20 Serviço de Infectologia e Controle de Infecção, Hospital Moinhos de Vento - Porto Alegre (RS), Brasil.; 21 Departamento de Medicina Interna, Universidade Federal do Rio Grande do Sul - Porto Alegre (RS), Brasil.; 22 Laboratório de Fisiopatologia Experimental, Programa de Pós-Graduação em Ciências da Saúde, Universidade do Extremo Sul Catarinense - Criciúma (SC), Brasil.; 23 Serviço de Medicina Intensiva, Hospital São José - Criciúma (SC), Brasil

**Keywords:** Coronavirus infections/drug therapy, COVID-19, SARS-CoV-2, Betacoronavirus, GRADE, Guidelines, Brazil, Infecções por coronavirus/tratamento farmacológico, COVID-19, Betacoronavirus, SARS-CoV-2, GRADE, Diretrizes, Brasil

## Abstract

**Introduction:**

Different therapies are currently used, considered, or proposed for the treatment of COVID-19; for many of those therapies, no appropriate assessment of effectiveness and safety was performed. This document aims to provide scientifically available evidence-based information in a transparent interpretation, to subsidize decisions related to the pharmacological therapy of COVID-19 in Brazil.

**Methods:**

A group of 27 experts and methodologists integrated a task-force formed by professionals from the Brazilian Association of Intensive Care Medicine (*Associação de Medicina Intensiva Brasileira -* AMIB), the Brazilian Society of Infectious Diseases (*Sociedad Brasileira de Infectologia* - SBI) and the Brazilian Society of Pulmonology and Tisiology (*Sociedade Brasileira de Pneumologia e Tisiologia -* SBPT). Rapid systematic reviews, updated on April 28, 2020, were conducted. The assessment of the quality of evidence and the development of recommendations followed the GRADE system. The recommendations were written on May 5, 8, and 13, 2020.

**Results:**

Eleven recommendations were issued based on low or very-low level evidence. We do not recommend the routine use of hydroxychloroquine, chloroquine, azithromycin, lopinavir/ritonavir, corticosteroids, or tocilizumab for the treatment of COVID-19. Prophylactic heparin should be used in hospitalized patients, however, no anticoagulation should be provided for patients without a specific clinical indication. Antibiotics and oseltamivir should only be considered for patients with suspected bacterial or influenza coinfection, respectively.

**Conclusion:**

So far no pharmacological intervention was proven effective and safe to warrant its use in the routine treatment of COVID-19 patients; therefore such patients should ideally be treated in the context of clinical trials. The recommendations herein provided will be revised continuously aiming to capture newly generated evidence.

## Abstract (pt)

**Introdução:**

Há diversas terapias sendo utilizadas, consideradas ou propostas para o tratamento da COVID-19, muitas carecendo de apropriada avaliação de efetividade e segurança. O propósito deste documento é fornecer recomendações baseadas nas evidências científicas disponíveis e em sua interpretação transparente, para subsidiar decisões sobre o tratamento farmacológico da COVID-19 no Brasil.

**Métodos:**

Um grupo de 27 especialistas e metodologistas integraram a força-tarefa formada pela Associação de Medicina Intensiva Brasileira (AMIB), pela Sociedade Brasileira de Infectologia (SBI) e pela Sociedade Brasileira de Pneumologia e Tisiologia (SBPT). Foram realizadas revisões sistemáticas rápidas, atualizadas até 28 de abril de 2020. A qualidade das evidências e a elaboração das recomendações seguiram o sistema GRADE. As recomendações foram elaboradas nos dias 5, 8 e 13 de maio de 2020.

**Resultados:**

Foram geradas 11 recomendações, embasadas em evidência de nível baixo ou muito baixo. Não há indicação para uso de rotina de hidroxicloroquina, cloroquina, azitromicina, lopinavir/ritonavir, corticosteroides ou tocilizumabe no tratamento da COVID-19. Heparina deve ser utilizada em doses profiláticas no paciente hospitalizado, mas não deve ser realizada anticoagulação na ausência de indicação clínica específica. Antibacterianos e oseltamivir devem ser considerados somente nos pacientes em suspeita de coinfecção bacteriana ou por influenza, respectivamente.

**Conclusão:**

Até o momento, não há intervenções farmacológicas com efetividade e segurança comprovada que justifiquem seu uso de rotina no tratamento da COVID-19, devendo os pacientes serem tratados preferencialmente no contexto de pesquisa clínica. As recomendações serão revisadas continuamente, de forma a capturar a geração de novas evidências.

## INTRODUCTION

Early in December 2019, the first cases of novel pneumonia from unknown etiology were described in the city of Wuhan, China. Subsequent studies have shown that it was caused by a new coronavirus, later called Severe Acute Respiratory Syndrome Coronavirus 2 (SARS-CoV-2).^(1,2)^ This infection caused by SARS-CoV-2 is called coronavirus disease 2019 (COVID-19), a new infectious disease affecting the respiratory tract and declared as a pandemic by the World Health Organization (WHO). The disease clinical features may range from asymptomatic or oligosymptomatic patients to severe respiratory failure cases requiring admission to the intensive care unit (ICU) and progression to Acute Respiratory Distress Syndrome (ARDS). In this context, the mortality may range between 26% and 86%.^(3,4)^ In Brazil, the first reported case of COVID-19 was recorded on February 25, 2020, in a patient returning from a trip to Italy. On May 21, 2020, Brazil had already 291,579 cases reported, and 18,859 deaths.^(5)^

In the scenery of a pandemic, a large portion of actions and interventions are empirical and based on findings usually derived from *in vitro* experiments, anecdotical personal experiences, and small observational studies lacking appropriate methodology. There is a relentless and, sometimes, an uncoordinated quest for potential therapy, and uncertainly effective drugs are fastly disseminated as potential savior therapies and globally included in treatment protocols. The medical decision-making process, usually guided by a rational evidence-based approach, becomes emotional. Although understandable from a social and humanitarian point of view in the context of a pandemic, this process can lead to over therapy with treatments used without an indication, and the consequent risk of adverse events.^(6-8)^ In such a context, guidelines are useful to guide healthcare professionals for a decision-making process based on the best evidence available.

This guideline for the treatment of COVID-19 patients is supported by the following medical societies: the Brazilian Association of Intensive Care Medicine *(Associação de Medicina Intensiva Brasileira -* AMIB*),* the Brazilian Society of Infectious Diseases *(Sociedade Brasileira de Infectologia -* SBI*)* and the Brazilian Society of Pulmonology and Tisiology *(Sociedade Brasileira de Pneumologia e Tisiologia -* SBPT*)*. Its main objective is to provide uniformity for therapeutic indications on the COVID-19 context and to guide these interventions utilizing the best evidence available at the time it is written.

## METHODS

This guideline was prepared according to the Grading of Recommendations Assessment, Development and Evaluation (GRADE) system for the assessment of evidence and development of the recommendations.^(9)^ An approach according to the Guidelines International Network and McMaster University (GIN/McMaster) for the development of rapid recommendations checklist was used.^(10)^ Our target audience is prescribing physicians who work in a public or private context, either in outpatient or hospital settings (including emergency departments and intensive care units).

### The guideline development group

The group consisted of 27 members, including physicians experts in infectious diseases, internal medicine, pulmonology, and intensive care, in addition to pharmacists, epidemiologists, and public health experts. The recommendation panel was composed of 13 voting members, guideline methodologists (five members), and researchers responsible for the literature systematic review (nine members). The participants were indicated by the specialty societies or by the group methodologists, aiming to provide representativity and balance of technical competencies.

Potential conflicts of interest data were collected with the standard WHO formulary. Members with a direct financial conflict of interest related to a given intervention had no right to vote for the related questions. A list of participants, their role in the guideline, and statement of conflicts of interest are provided in appendix 1.

### Research questions

The questions were first proposed by the group of methodologists, and revised by the panel experts. The inclusion criteria were: a drug available for prescription in Brazil, and a clinical practice variability, or a clinically relevant doubt related to its use, being this last the prioritizing factor for clinical questions.

Eight questions were prepared according to the acronym PICO (population, intervention, comparator, and outcome), considering six drug classes (aminoquinolines, antivirals, antibiotics, corticosteroids, anticoagulants, and immunobiologicals). Each research question could generate one or more recommendations.

Remdesivir was not included in this document as, by the assessment time, it was not approved for prescription in Brazil. Were considered, but not prioritized, questions related to the use of nonsteroidal anti-inflammatory drugs and angiotensin receptor blockers (ARBs) or angiotensin-converting enzyme inhibitors. During the conduction of the recommendations panel, the question on the use of heparin was requested to be included, once it was not part of the initial scope.

### Evidence search and synthesis

The searches were conducted on MEDLINE (via PubMed®), the Cochrane CENTRAL, and Embase databases, and also in the grey-literature bases, between April 22 and 30, 2020. Additionally searches on prepublication articles sources, such as OpenGrey (http://www.opengrey.eu), meDrxiv (https://www.medrxiv.org), and Biorxiv (www.biorxiv.org) were conducted; these articles were considered during the analyses, even though not peer-reviewed. The search strategies are shown in appendix 2. Additional evidence, emerging during the process and identified by members of the group, were considered during the discussions, although not included in the initial search results.

Literature search, data extraction, and synthesis were performed by one single investigator and checked by a second revisor in case of doubts or inconsistencies.^(11,12)^ The synthesis was conducted in a qualitative model. First, titles and abstracts of the identified manuscripts were identified by the search strategy, and potentially eligible studies preselected. Second, the full text of the selected papers was assessed, to confirm eligibility.

Considering the limited number of studies published so far, the following study designs were taken into consideration according to the evidence hierarchy: randomized clinical trials, quasi-randomized clinical trials, non-randomized clinical trials, cohort studies, case-control studies, series and case studies. The assessment of methodological quality and/or risk of bias of the included articles was conducted with tools appropriate for each study design: AMSTAR-2 for systematic reviews with and without metanalysis;^(13)^ Cochrane’s risk of bias table for randomized clinical trials;^(14)^ ROBINS-I for non-randomized or quasi-randomized clinical trials;^(15)^ ROBINS-I or Newcastle-Ottawa for longitudinal comparative observational studies (case-control and cohort);^(15,16)^ the Joanna Briggs Institute case-series tool for phase I or phase II trials without a direct comparator and case-series^(17)^ and the Joanna Briggs Institute toll for cross-sectional studies.^(18)^

### Assessment of certainty of the evidence and development of recommendations

The GRADE^(9,19)^ system was used for the assessment of the quality of the evidence and the development of recommendations. Previously to the recommendations meetings, the certainty of the evidence was rated either as high, moderate, low, or very low (Table 1).

**Table 1:** Levels of Evidence according to the Grading of Recommendations Assessment, Development, and Evaluation (GRADE)

Level	Definition	Implications
High	Strong confidence that the true effect lies close to that of the effect estimate.	It is unlikely that additional trials will change the confidence in the estimated effect.
Moderate	Moderate confidence in the effect estimate.	Future trials may modify the confidence in the effect estimate, and also can change the estimate.
Low	Limited confidence in the effect estimate.	Future trials are likely to importantly impact our confidence in the effect estimate.
Very Low	Uncertain confidence in the effect estimate.	Any effect estimate is uncertain.

Source: Grading of Recommendations Assessment, Development, and Evaluation (GRADE) Working Group. Handbook for grading the quality of evidence and the strength of recommendations using the GRADE approach. Updated October 2013. Available from: https://gdt.gradepro.org/app/handbook/handbook.html.^(19)^

According to this methodology, recommendations may be either strong or weak (conditionals), favorable or against the intervention. The implication of the strength of the recommendation is shown in table 2.

**Table 2:** Strength of recommendation according to the Grading of Recommendations Assessment, Development, and Evaluation (GRADE)

Target audience	Strong	Weak (conditional)
Policymakers	The recommendation should be adopted as a healthcare policy in most of the situations.	Substantial debate required and the involvement of stakeholders.
Clinicians	Most of the individuals would want the intervention to be indicated, and only a small number would reject the recommendation.	A large portion of the individuals would want the intervention to be indicated; however, some individuals would reject the recommendation.
Patients	Most of the patients should receive the recommended intervention.	The clinician should acknowledge that different choices are appropriate for each patient and choose consistently with his/her values and preferences.

Source: Grading of Recommendations Assessment, Development, and Evaluation (GRADE) Working Group. Handbook for grading the quality of evidence and the strength of recommendations using the GRADE approach. Updated October 2013. Available from: https://gdt.gradepro.org/app/handbook/handbook.html.^(19)^

The data from the systematic review for each PICO question were compiled into evidence profiles and presented to the experts’ panel.^(20-24)^ For the development of recommendations were considered: benefits, risks, quality of the evidence, costs, and variability of implementation. The recommendations were agreed upon by teleconferences held on May 5, May 8, and May 13, 2020.

Whenever appropriate, the panel could provide recommendations according to subpopulations. A consensus was aimed for all the recommendations; if no consensus could be achieved, a voting procedure was conducted, and a simple majority required for approval of that specific recommendation.

### Population of interest

The target population for the recommendations is constituted of patients either diagnosed or clinically suspected of SARS-CoV-2 infection. Clinical suspicion is when, based on epidemiological data, clinical history, signs, and symptoms, in addition to complementary tests, COVID-19 is the most likely diagnostic hypothesis. Information on diagnosis can be found elsewhere.^(25-27)^

The disease severity was grouped into five categories, in line with the National Institutes of Health (NIH) guidelines for the treatment of COVID-19 (Table 3).^(28)^ For the categorization of acute respiratory distress syndrome (ARDS) the Berlin’s criteria were adopted as presented in table 4.

**Table 3:** Classification of COVID-19 severity

Classification	Description
Asymptomatic or presymptomatic infection	A positive test for SARS-CoV-2, without symptoms
Mild disease	Signs or symptoms (e.g.: fever, coughing, fatigue, muscle pain, and headache), but no shortness of breath, dyspnea or abnormal image tests
Moderate disease	Evidence of lower respiratory tract disease (either clinical or image tests) and SpO_2_ > 93% breathing room air
Severe disease	Presence of one of these features:
	- Respiratory rate > 30 breaths per minute
	- SpO_2_ ≤ 93% breathing room air
	- PaO_2_/FiO_2_ < 300
	- Pulmonary infiltrate > 50%
Critical disease	Respiratory failure, septic shock, and/or multiple organ failure

SARS-CoV-2 - severe acute respiratory syndrome 2; SpO_2_ - oxygen saturation; PaO_2_/FiO_2_ - partial pressure of oxygen/fraction of inspired oxygen.

Source: adapted from National Institutes of Health. Treatment Guidelines Panel. Coronavirus Diseases 2019 (COVID-19). COVID-19 Treatment Guidelines. [cited 2020 May 18]. Available from:  https://www.covid19treatmentguidelines.nih.gov/.^(28)^

**Table 4:** Classification of acute respiratory distress syndrome

	Mild	Moderate	Severe
Start time	Sudden manifestation within one week after exposure to a risk factor or appearance or worsening of respiratory symptoms		
Hypoxemia - PaO_2_/FiO_2_	201 - 300 with PEEP/CPAP/EPAP ≤ 5	101 - 200 with PEEP/CPAP/EPAP ≤ 5	≤ 100 with PEEP/EPAP ≤ 5
			
Edema origin	Not clearly explained respiratory failure due to heart failure or volume overload		
Radiological abnormalities	Bilateral opacities represented not only by nodes, effusions, masses or lobar/pulmonary collapses;		

PaO_2_/FiO_2_ - partial pressure of oxygen/fraction of inspired oxygen; PEEP - positive end-expiratory pressure; CPAP - continuous positive airway pressure; EPAP - expiratory positive airway pressure.

Source: adapted from Associação de Medicina Intensiva Brasileira, Sociedade Brasileira de Pneumologia e Tisiologia. Diretrizes Brasileiras de Ventilação Mecânica 2013. São Paulo: AMIB; 2013. [citado 2020 Abr 19]. Disponível em https://www.amib.org.br/fileadmin/user_upload/amib/2018/junho/15/Diretrizes_Brasileiras_de_Ventilacao_Mecanica_2013_AMIB_SBPT_Arquivo_Eletronico_Oficial.pdf.^79^

## RESULTS

Eleven recommendations were issued. These recommendations are summarized in table 5. Detailed information on the evidence is shown in appendix 3, as GRADE evidence profiles, with complete references.

**Table 5:** Summary of recommendations

**Recommendation 1**: we suggest against the routine use of hydroxychloroquine or chloroquine for treatment of COVID-19 patients (weak recommendation; Level of Evidence low)
**Recommendation 2:** we suggest against the routine use the hydroxychloroquine or chloroquine plus azithromycin combination for treatment of COVID-19 patients (weak recommendation; Level of Evidence very low)
**Recommendation 3:** we recommend against the use of oseltamivir for the treatment of COVID-19 in patients no suspected influenza coinfection (strong recommendation; Level of Evidence very low)
**Recommendation 4:** we suggest the use of empirical oseltamivir treatment in patients with severe acute respiratory syndrome or flu-like syndrome with risk factors for complications when a diagnosis of influenza cannot be ruled out (weak recommendation; Level of Evidence very low)
**Recommendation 5:** we suggest against the routine use lopinavir/ritonavir for treatment of COVID-19 (weak recommendation; Level of Evidence low)
(recommendation weak; Level of Evidence very low)
**Recommendation 7:** we suggest against the routine use tocilizumab for COVID-19 treatment (weak recommendation; Level of Evidence very low)
**Recommendation 8:** we recommend the routine use of venous thromboembolism prophylaxis in COVID-19 hospitalized patients (strong recommendation; Level of Evidence very low)
**Recommendation 9:** we suggest against the routine use therapeutic heparin doses for COVID-19 treatment (weak recommendation; Level of Evidence very low)
**Recommendation 10:** we suggest against the use prophylactic antibiotics in patients with a suspected or confirmed COVID-19 diagnosis (weak recommendation; Level of Evidence very low)
**Recommendation 11:** we recommend the use of antibiotics in COVID-19 patients with suspected bacterial coinfection (recommendation not rated)

### Aminoquinolines (hidroxychloroquine and chloroquine)

**Recommendation 1** - we suggest against the routine use of hydroxychloroquine or chloroquine for treatment of COVID-19 patients (**weak recommendation; Level of Evidence: low**).

**Summary of the evidence**: the systematic review identified three comparative clinical trials with available data on the effects of hydroxychloroquine (HCQ) in COVID-19 patients: two open randomized clinical trials^(29,30)^ in a patient population with mild to moderate disease, and one cohort study.^(31)^ No trials comparing chloroquine (CQ) to non-CQ therapy were found. The combined data from both clinical trials failed to show clinical-radiological improvement (relative risk - RR = 0.61; 95% confidence interval [95%CI] 0.26 - 1.43), or improved viral negativation rates within seven days (RR = 2.00; 95%CI 0.02 - 20.00),^(29,31)^ however, one of the studies prepublication version has shown on the raw data analysis an increased improvement rate with HCQ (80.6% *versus* 54.8%; p = 0.0476).^(30)^ Mortality and mechanical ventilation requirements, considered to be clinically relevant outcomes, were assessed in an observational study with 364 patients,^(32)^ showing an increased mortality rate with HCQ (HCQ: 27.8%; HCQ + azithromycin: 22.1%; standard therapy: 11.4%), with a significant association remaining after the propensity score-adjusted analysis comparing HCQ to the standard therapy (hazard ratio - HR = 2.61; 95%CI 1.10 - 6.17).^(32)^ After the review date, an additional cohort study was identified involving 1,376 moderate to severe COVID-19 hospitalized patients. In this study, no association was found with death or invasive mechanical ventilation requirement (HR = 1.04; CI95% 0.82 - 1.32).^(33)^ This trial was considered in the evidence analysis and because it was a well-designed observational study, with adjustment for confounders and appropriate sample size, the degree of confidence for the lack of benefit was increased from very low, to low.

The association between HCQ and arrhythmias is well-known. An observational study has shown that seven out of 37 (19%) patients receiving HCQ monotherapy developed a QT interval ≥ 500ms.^(34)^ Additionally, in a randomized clinical trial that compared patients using high-dose (1,200mg CQ for 10 days; total dose 12g) *versus* a lower dose (900mg on the first day, followed by 450mg daily for four days; total dose 2.7g), a 13.5% (95%CI 6.9 - 23.0%) overall mortality rate was found in association with the high-dose (in this trial both groups had a cointervention with ceftriaxone and azithromycin), suggesting a potential dose-response gradient.^(35)^

**Comments**: the recommendations panel interpreted that the available evidence suggests no clinically significant benefit of HCQ or CQ therapy. There was an agreement that the risk of cardiovascular adverse events is moderate, particularly regarding arrhythmias. So far, the existing comparative trials have only assessed hospitalized patients, therefore providing no basis for considerations on using or not these drugs in outpatients. The prescription of these products may be considered on a shared clinician-patient decision, only for severe or critically ill patients, hospitalized, under constant QTc interval monitoring, and avoiding concomitant QTc prolonging therapies. Its use should preferably be under clinical trial protocols.

### Aminoquinolines (hydroxychloroquine and chloroquine) in association with azithromycin

**Recommendation 2** - we suggest against the routine use the hydroxychloroquine or chloroquine plus azithromycin combination for treatment of COVID-19 patients (**weak recommendation; Level of Evidence very low**).

**Summary of the evidence:** no clinical trials were identified to assess azithromycin monotherapy. Azithromycin added to an HCQ regimen was assessed in one single trial, showing improved viral negativation in the group treated with the combination therapy (HQ + azithromycin 100%; n = 6/6 *versus* HQ 57%; n = 8/14; six-day negativation).^(36)^ No randomized clinical trials were identified for the comparison of HCQ + azithromycin *versus* standard therapy. Viral negativation was assessed by four studies, three from the same research group, showing viral negativation above 90% after five to 10 days therapy;^(36-38)^ in contrast, in one study treating ten patients with HCQ + azithromycin, negativation was found in only two patients (20%).^(39)^ A total of six studies assessed mortality, with 35 deaths among 1,342 patients.^(35,37-41)^

Regarding adverse cardiovascular events, eight studies were identified. Five of them found a prolonged QT interval in some patients treated with HCQ/CQ plus azithromycin.^(35,39,40,42,43)^ A retrospective analysis of 130 thousand rheumatoid arthritis patients showed an increased risk of cardiovascular death with HCQ + azithromycin as compared with HCA + amoxicillin (HR = 2.19; 95%CI 1.22 - 3.94; 30-day outcome). ^(44)^ The same analysis has also shown an increased risk of angina (HR = 1.15; 95%CI 1.05 - 1.26) and heart failure (HR = 1.22; 95%CI 1.02 - 1.45). In a non-comparative trial with 1,061 patients with HCQ + azithromycin, no patient has shown to have heart toxicity.^(38)^

**Comments**: the recommendations panel interpreted that the available evidence does not suggest a clinically significant benefit from the treatment with HCQ or CQ in combination with azithromycin. There was an understanding that there is an associated moderate increase of cardiovascular adverse events, especially arrhythmias, potentialized by the association of HCQ/CQ with azithromycin, and additional care related to these adverse events is required. So far, the existing comparative trials have only assessed hospitalized patients, providing no basis for discussions on using or not this combination in outpatients. Its use may be considered in a shared clinician-patient decision, only in severe or critically ill patients, hospitalized, with frequent QTc interval monitoring and avoiding QTc prolonging concomitant therapy. Its use should be preferentially under clinical trial protocols.

### Oseltamivir

**Recommendation 3** - we recommend against the use of oseltamivir for the treatment of COVID-19 in patients with no suspected influenza coinfection **(strong recommendation**; **Level of Evidence very low**).

**Recommendation 4 -** We suggest the use of empirical oseltamivir treatment in patients with Severe Acute Respiratory Syndrome (SARS) or flu-like syndrome with risk factors for complications when a diagnosis of influenza cannot be ruled out (**weak recommendation; Level of Evidence very low**).

**Summary of the evidence:** no randomized clinical trial assessing the effectivity of oseltamivir in COVID-19 patients was identified. A cohort study with 504 COVID-19 hospitalized patients assessed the use of oseltamivir, lopinavir/ritonavir, and umifenovir.^(45)^ The mortality rate in the oseltamivir group (n = 66) was 12.2% *versus* 16.2% in the non-oseltamivir group (odds ratio - OR = 0.71; 95%CI 0.28 - 1.59). Also, no difference was found regarding lung injury improvement as assessed by a chest CT scan (41.2% *versus* 43.3%). The study has important methodological issues, such as lacking randomization, sample representativity, and control for confounders.

**Comments:** the recommendations panel interpreted that there is no evidence to support the use of oseltamivir for SARS-CoV-2 therapy; besides, there is no theoretical rationale to support this use.

However, oseltamivir may be considered in cases of suspected influenza infection in patients with ARDS or flu-like syndrome with risk-factors for influenza complications (chronic diseases, immunosuppression, age ≥ 65 years, and pregnant women).^(46)^ The usual dose for adults with appropriate renal function is 75mg twice daily for five days.^(25,47)^ Suspected influenza should take into consideration the patient’s symptoms, radiological findings, as well as local epidemiology. A suspect may remain even in individuals with a history of immunization, once the vaccine’s effectiveness is rarely above 80%.^(48,49)^ If testing for influenza is possible, oseltamivir may be stopped upon negative results, given the available test has appropriate sensitiveness for seasonal A, B, and H1N1 influenza. The decision on oseltamivir use was made based on indications for its use out of the context of the COVID-19 pandemic, with no appropriate information on the behavior of influenza in the scenery of a SARS-CoV-2 epidemics.^(46)^ If well-developed local protocols are in place, we suggest them to be adhered to.

### Lopinavir/ritonavir

**Recommendation 5 -** we suggest against the routine use lopinavir/ritonavir for treatment of COVID-19 (**weak recommendation; Level of Evidence low**).

**Summary of the evidence -** two randomized clinical trials assessed the use of lopinavir/ritonavir in COVID-19 patients.^(50,51)^ One of them assessed 90 patients in the lopinavir/ritonavir group and 100 patients with standard therapy. Patients in the intervention group had lower but not statistically significant mortality rates (19.2% *versus* 25%; 28-day mortality rate), and no clinically significant improvement within 14 days (45.5% with the intervention *versus* 30% in the control group; p < 0.05); the time to clinical improvement was reduced in one day (median: 15 days *versus* 16 days; HR = 1.39; 95%CI 1.00 - 1.91; modified intention-to-treat analysis, excluding three early mortality patients).^(50)^ Another randomized clinical trial included 21 patients in the lopinavir/ritonavir group and seven in the control group, with no statistically significant difference between the groups for outcomes such as fever, coughing relief rate, clinical condition deterioration rate, and CT scan improvement.^(51)^ In both trials, there was no viral negativation difference between the groups. The adverse effects observed included anorexia, nausea, abdominal discomfort or diarrhea, acute gastritis, and reduced appetite; the lopinavir/ritonavir discontinuation rate was 13.8%.^(5l)^

**Comments:** the recommendations panel interpreted that the available evidence suggests no clinically significant benefit from the lopinavir/ritonavir therapy. This therapy could be considered promising, and the lack of observed benefits may result from the small number of assessed patients. Despite the high discontinuation rate due to adverse events and potential drug interactions, lopinavir/ritonavir is a relatively safe therapy for short term courses. This drug may be considered upon a clinician-patient shared decision, in hospitalized severe and critically ill patients, in centers with professionals experienced with this therapy. It should be preferably used under clinical trial protocols.

### Corticosteroids

**Recommendation 6 -** we suggest against the routine use corticosteroids for COVID-19 patients’ treatment (**weak recommendation; Level of Evidence very low**).

**Summary of the evidence:** no clinical trials specifically assessing the use in COVID-19 patients were found. Four observational studies reported that the use of corticosteroids during hospitalization is associated with increased mortality; these studies combined hospitalized patients’ populations, however, with heterogeneous clinical features.^(52-55)^ One trial, however, suggests that the use of methylprednisolone reduced the risk of death in patients with ARDS (HR = 0.38; 95%CI 0.20 - 0.72).^(56)^ The outcomes for respiratory symptoms are variable in hospitalized patients.^(57,58)^ The studies’ limitations include the lack of randomization and control groups, variable doses used, small samples, and retrospective analysis of the data.

Despite the systematic review conducted did not involve other coronaviruses infections, indirect information on SARS and Mid-East respiratory syndrome (MERS) show an absence of impact on mortality (RR = 1.07; 95%CI 0.81 - 1.42) and a prolonged time to viral negativation (3.78 days; 95%CI 1.16 - 6.41 days).^(59)^

**Comments:** the recommendations panel interpreted that there is no evidence supporting the routine use of corticosteroids for COVID-19 patients. Corticosteroids should be avoided during the first seven to 10 days after the symptoms start, when the viral response is more relevant, as there is evidence that corticosteroids may delay viral negativation.

Some evidence points out to a potential benefit for moderate to severe ARDS patients out of the viral infection context.^(60)^ Its use may be considered for selected cases with moderate to severe ARDS, without suspected uncontrolled bacterial infection, 10 to 14 days after the COVID-19 symptoms start. The doses used in the studies ranged between 10mg and 20mg dexamethasone or 40mg to 120mg methylprednisolone daily, for five to 10 days. Their use should preferentially be under clinical research protocols.

Patients with other indications for corticosteroids use (e.g.: asthma and exacerbated chronic obstructive pulmonary disease - COPD) should use these drugs according to the clinical indication, assessing other potential risks and benefits during COVID-19 infection.

### Tocilizumab (anti-interleukin-6)

**Recommendation 7 -** we suggest against the routine use tocilizumab for COVID-19 treatment (**weak recommendation; Level of Evidence very low**).

**Summary of the evidence:** no comparative trials evaluating tocilizumab effectiveness in COVID-19 patients were found; only two case series were identified. One of the series included 21 patients, all with chest CT changes, 20 on ventilatory support (45% with high-flow oxygen, 35% with a nasal cannula, 5% with an oxygen mask, 5% with non-invasive mechanic ventilation and 10% with invasive mechanical ventilation). Within five days, 75% of them had their ventilatory support requirements reduced; no deaths occurred during the follow-up period.^(61)^ In another case series 15 patients were included, two moderately ill, six with severe disease, and seven in a very severe condition. Of the 15 patients, three died, two had increased severity, nine had clinical stabilization and one showed clinical improvement. Serum interleukin-6 (IL-6) was reduced in 10 patients after tocilizumab; increased IL-6 was found in the five patients with treatment failure, all of them with an initially very severe condition.^(62)^

**Comments:** the recommendation panel interpreted that no benefit and safety evidence was shown that would suggest the routine use of tocilizumab. Besides, this drug is costly, and especially during an epidemic, the use of resources should be rationalized, and the use of interventions with no benefit evidence avoided. This drug may be considered in a shared clinician-patient decision for severe and critically ill hospitalized patients, with a confirmed diagnosis of SARS-CoV-2 infection, and significantly increased markers or inflammation (e.g.: IL-6, D-dimer, C reactive protein, lactate dehydrogenase - LDH, and ferritin). The use of tocilizumab should be restricted to centers with professionals who are experienced in its use. Tocilizumab use should preferentially be under clinical trial protocols.

### Heparins

**Recommendation 8 -** we recommend the routine use of venous thromboembolism prophylaxis in COVID-19 hospitalized patients (**strong recommendation; Level of Evidence very low**).

**Recommendation 9 -** we suggest against the routine use therapeutic heparin doses for COVID-19 treatment (**weak recommendation; Level of Evidence very low**).

**Summary of the evidence:** two retrospective cohorts were identified.^(63,64)^ One of them assessed 449 severe or critically ill hospitalized COVID-19 patients; 99 of them were given heparin for at least seven days (94 enoxaparin 40mg - 60mg daily and five unfractionated heparin 10,000 - 15,000IU/daily), and 350 control patients (without any anticoagulant or heparin, or less than seven days of use). In this study, the 28-day mortality rate was similar (heparin 30.3% *versus* controls 29.7%). In the subgroup with an International Society on Thrombosis and Hemostasis - Sepsis-Induced Coagulopathy score (ISTH SIC) ≥ 4 (from one of the following features: platelets < 100,000, International Normalized Ratio - INR > 1.4 or a Sequential Organ Failure Assessment - SOFA - score ≥ 2), the mortality in the heparin group was lower (40% *versus* 64.2%; p = 0.029; n = 97). Increased effectiveness was also seen in patients with increased D-dimer, with a significantly reduced mortality in a group with D-dimer values above or equal to six-times the upper limit of the normal (32.8% *versus* 52.4%; p = 0.017).^(64)^ In a study that assessed 42 patients, all of them with an immunosuppressor or corticosteroids, and severe to moderate COVID-19, 21 patients were given low molecular weight heparin (13 enoxaparin 40mg daily, two enoxaparin 20mg daily, four nadroparin 4,100IU daily and two low molecular weight sodium heparin 5,000IU daily, median: 11 days) and 21 controls, D-dimer and IL-6 levels were significantly reduced, and lymphocytes counts were increased, with no hospital length of stay differences.^(65)^

**Comments:** the recommendations panel interpreted that there is no indication for therapeutic dose heparins (e.g.: enoxaparin 1mg/kg subcutaneously - SC - every 12 hours) for the treatment of COVID-19. The rationale for other anticoagulants is analogous. Anticoagulation is associated with an increased risk of bleeding events and should be restricted to patients with a clear indication (e.g.: atrial fibrillation, pulmonary thromboembolism, deep venous thrombosis, among others), according to appropriate protocols.

COVID-19 patients apparently have an increased risk of thromboembolic events, and the assisting team should be aware of developing signs and symptoms. COVID-19 hospitalized patients should be given thromboembolism prophylaxis according to risk-stratification strategies, adhering to local hospital protocols. However, the use of prophylactic doses can be extended to all COVID-19 patients, as some SARS-CoV-2 patients appear to have a hypercoagulability state, with increased thromboembolic events rate as seen in observational clinical trials and *post mortem* examinations.^(66,67)^ As an example, enoxaparin 40mg to 60mg SC once-daily doses, or unfractionated heparin 5,000IU SC twice or three times a day, could be used. Although there is limited evidence for pharmacological prophylaxis in COVID-19 patients, this is a low-cost and well-tolerated intervention that may potentially prevent major clinical events. Heparin should not be used for cases with contraindications (e.g.: increased risk of bleeding, active bleeding, and severe thrombocytopenia);^(68)^ low molecular weight heparin should be used carefully in renal dysfunction patients.

### ANTIBIOTICS

**Recommendation 10 -** we suggest against the use prophylactic antibiotics in patients with a suspected or confirmed COVID-19 diagnosis (**weak recommendation; Level of Evidence very low**).

**Recommendation 11 -** we recommend the use of antibiotics in COVID-19 patients with suspected bacterial infection (**non-rated recommendation**).

**Summary of the evidence**: no randomized clinical trials were found to assess the empirical antibiotics therapy effectiveness in COVID-19 patients without evidence of bacterial infections. Therefore, so far there is no clinical data enough to show benefits or risks of antibiotics in COVID-19 patients with no signs of bacterial infection. We did not access the evidence for bacterial infections therapy.

**Comments:** the panel interpreted that considering the lack of evidence, there is no base for prophylactic antibiotic therapy in COVID-19 patients. In addition to the lack of benefit evidence, this could result in adverse events, increased antimicrobial resistance, and costs.

There is no appropriate data on bacterial coinfection in COVID-19 patients, however, one should bear in mind that overlapping infections may occur. It is understood that those patients should be given antibiotics, similarly to COVID-19 patients, taking into consideration the local epidemiology and adhering to local protocols and guidelines from infection control services.

Table 6 shows a didactic summary of the recommendations according to the evaluated interventions, presenting their judgment regarding perceived benefits, risks, costs, availability, and evidence. Appendix 4 presents the most important drug interactions of potential COVID-19 therapies.

**Table 6:** Summary of recommendations and judgments

Intervention	Benefit	Risk	Cost*	Access	Evidence^†^	Recommendation
Pharmacological therapy for COVID-19						
Hydroxychloroquine (or chloroquine)	Small or neglectable	Important	Low	Good^‡^	Low	Against routine use (weak)
Hydroxychloroquine (or chloroquine) + azithromycin	Small or neglectable	Important	Low	Good^‡^	Very low	Against routine use (weak)
Lopinavir/ritonavir	Small or neglectable	Moderate	Low	Limited^§^	Low	Against routine use (weak)
Oseltamivir	Small or neglectable	Small or neglectable	Low	Good^‡^	Very low	Against the use (strong)
Tocilizumab	Small or neglectable	Moderate	High	Limited^§^	Very low	Against routine use (weak)
Corticosteroids	Small or neglectable	Important	Low	Good^‡^	Very low	Against routine use (weak)
Heparin - anticoagulant dose	Small or neglectable	Important	Moderate	Good^‡^	Very low	Against routine use (weak)
Conditions associated with COVID-19						
Oseltamivir (suspected influenza in severe cases or risk factors)	Moderate	Small or neglectable	Low	Good^‡^	Very low	Favorable to use (weak)
Prophylactic heparin doses (hospitalized patients)	Moderate	Small or neglectable	Low	Good^‡^	Very low	Favorable to use (strong)
Antibiotics (prophylactic)	Small or neglectable	Small or neglectable	Low	Good^‡^	Very low	Against the use (weak)
Antibiotics (suspected bacterial infection)	Important	Small or neglectable	Low	Good^‡^	Not assessed	Favorable to use

Qualitative assessment, considering public and complementary health systems, based on prices according to the *Painel de Preços do Ministério da Economia* (Ministry of Economy Prices Panel), *Banco de Preços em Saúde* (Health Products Prices Data Bank), *Tabela Câmara de Regulação do Mercado de Medicamentos* (Table from the Medicines Price Regulation Chamber) and usual market prices;

† evidence assessed according to the Grading of Recommendations Assessment, Development, and Evaluation (GRADE);

‡ good availability in the Brazilian context, either products or use experienced professionals.

## DISCUSSION

During epidemics, when consolidated effective therapies are not available, there is a trend to use therapies based on preclinical studies results, or based on observational studies with important limitations.^(69)^ Experiences from other epidemics have shown that such interventions benefits may be far below the expected, as with oseltamivir during the influenza A (H1N1) epidemics in 2009.^(60,70)^ During the Ebola virus epidemics in 2014, several interventions were tested, including CQ, HCQ, favipiravir, immunobiological agents, and convalescent plasma; none was proven effective.^(71)^

By the time when this guideline is publicized, we face a scenario where no specific COVID-19 proposed intervention is proven effective. Regarding safety, drugs such as HCQ (especially when combined with azithromycin) at the doses proposed for COVID-19 have been shown relevantly associated with cardiovascular events.^(34,35,40,45)^ In the absence of effective therapies, treatment under clinical trial protocols should be encouraged. In this context, healthcare professionals should seek information on therapeutic clinical trials, especially randomized clinical trials, already approved by regulatory agencies and ethical committees possibly ongoing in their institutions.

Several other interventions are proposed, such as remdesivir, beta interferon, ivermectin, nitazoxandine, convalescent plasma, umifenovir, among others.^(72-77)^ This guideline chose to prioritize those interventions raising more clinical practice concerns in Brazil by the time of its development. Of note, the current speed of COVID-19 knowledge generation renders these recommendations prone to become outdated in a short frame of time. As most of the interventions are based on evidence from small observational or interventional trials, we understand that as new well-designed clinical trials, with appropriate sample sizes, are published, there is a huge potential that herein presented recommendations shall be changed. Therefore, it is of paramount importance that readers of this guideline keep this in mind as one of the most important limitations of this document.

Also, it is necessary to understand that a clinical guideline aims to guide the clinical practice not necessarily applicable to every patient. The scarcity of evidence with appropriate methodology renders impossible to provide more categorical recommendations; we stress that a significant portion of the studies we evaluated was preliminary published in Ahead of Print bases, with no editorial board and peer review evaluations. Therefore, in this document we present suggested actions, to be contextualized according to features such as the patient’s clinical profile, existing comorbidities, and risk of developing adverse effects, in addition to the assisting team’s experience with the proposed interventions, patient’s preferences, service structure, as well as costs and available resources. Regarding costs, in the context of public health, it is important to emphasize that, in a scenario of an epidemic, resource allocation should be prioritized to interventions more likely to be beneficial, such as Personal Protective Equipment and interventions related to the patient’s ventilatory support. Therefore, under the light of the current COVID-19 knowledge, some investments in pharmacological therapies are debatable. However, the treatment of patients under clinical trial protocols, with appropriate study design and the potential to provide an answer to the society, should be encouraged.

With this document we hope to guide clinical practice in a national context, therefore reducing the therapeutic variability. In addition to the evidence available in the scientific literature, the recommendations took into consideration some Brazilian specific features, such as the availability of some drugs (either because of lack of regulatory clearance or due to difficult access), population and healthcare professionals acceptance, and costs associated with their use. Also, most of this document’s recommendations are in line with WHO therapeutic recommendations.^(27)^ This document consists of a joint positioning by three medical societies, taking into consideration the need for the development of encompassing recommendations and contextualization of different medical specialties regarding of the frailty of the available evidence, that may be an applicable tool both for physicians working in the public health system and the supplementary system.

The developing group is committed to strive for periodically bring updates to this document, in a context of living guidelines,^(78)^ where recommendations are updated as new evidence becomes available. Additional interventions will be included as they become relevant doubts for COVID-19 therapy.

## Apêndice 1 - Potenciais conflitos de interesses dos participantes da diretriz

Data: 5 de maio de 2020

A lista abaixo é referente aos potenciais conflitos de interesses (CI) do grupo de painelistas e metodologistas relacionados a esta diretriz. A avaliação dos possíveis CI foi determinada por um processo de revisão pelo grupo coordenador do documento. A declaração de potenciais CI foi realizada por meio do formulário da Organização Mundial da Saúde (https://www.who.int/about/ethics/declarations-of-interest).

**Table:**

Participante	Potencial conflito de interesse	Participação
Alexandre Zavascki	Relacionados à diretriz: membro do comitê diretivo dos estudos COALIZÃO (não remunerado), avaliando hidroxicloroquina,azitromicina, corticoesteroides e tocilizumabe, financiado pelo Ministério da Saúde, EMS e hospitais filantrópicos Não relacionados à diretriz: recebeu financiamento de pesquisa da Pfizer	Painelista
Amilton Silva Júnior	Não há conflitos de interesse	Painelista
Angela Bagattini*	Não há conflitos de interesse	Revisão sistemática
Bruno Tavares	Não há conflitos de interesse	Painelista
Cassia Garcia Moraes Pagano*	Não há conflitos de interesse	Revisão sistemática
Cinara Stein*	Não há conflitos de interesse	Metodologista da diretriz Revisão sistemática
Clovis Cunha	Não relacionados à diretriz: Sociedade Brasileira de Infectologia (SBI)	Painelista
Daniela Pachito	Não há conflitos de interesse	Revisão sistemática
Débora Graf*	Não há conflitos de interesse	Revisão sistemática
Felipe Dal Pizzol	Não relacionados à diretriz: Associação de Medicina Intensiva Brasileira (AMIB)	Painelista
Flávia Medeiros*	Não há conflitos de interesse	Revisão sistemática
Gabriela Brito*	Não há conflitos de interesse	Revisão sistemática
Haliton Oliveira Júnior*	Relacionados à diretriz: fornecimento de medicação pela EMS para pesquisa sobre hidroxicloroquina (pesquisador no estudo COALIZÃO 5)	Metodologista Revisão sistemática
José Chatkin	Não relacionados à diretriz: Sociedade Brasileira de Pneumologia e Tisiologia (SBPT)	Painelista
Jessica Matuoka	Não há conflitos de interesse	Revisão sistemática
Lays Marra*	Não há conflitos de interesse	Revisão sistemática
Leandro Fritscher	Não relacionados à diretriz: Sociedade Brasileira de Pneumologia e Tisiologia (SBPT)	Painelista
Luciano Azevedo	Relacionados à diretriz: membro do comitê diretivo dos estudos COALIZÃO (não remunerado), avaliando hidroxicloroquina, azitromicina, corticoesteroides e tocilizumabe, financiado pelo Ministério da Saúde, EMS e hospitais filantrópicos Não relacionados à diretriz: recebeu financiamento de pesquisa da Aché Indústria Farmacêutica	Painelista
Maicon Falavigna*	Relacionados à diretriz: membro do comitê diretivo dos estudos COALIZÃO (não remunerado), avaliando hidroxicloroquina, azitromicina, corticoesteroides e tocilizumabe, financiado pelo Ministério da Saúde, EMS e hospitais filantrópicos. Membro do GRADE Working Group Não relacionados à diretriz: sócio da empresa de consultoria HTAnalyze, tendo executado e recebido honorários de projetos com Roche, PTC Therapeutics, Sanofi, Boehringer e AbbVie. Sócio da empresa de teleconsultoria em saúde INOVA Medical	Metodologista da diretriz Revisão sistemática Coordenador
Marcelo Gazzana	Relacionados à diretriz: membro do comitê diretivo dos estudos COALIZÃO (não remunerado), avaliando hidroxicloroquina,azitromicina, corticoesteroides e tocilizumabe, financiado pelo Ministério da Saúde, EMS e hospitais filantrópicos.	Painelista
Michele Nunes	Não relacionados à diretriz: Associação de Medicina Intensiva Brasileira (AMIB)	Painelista
Mirian Dalben	Relacionados à diretriz: pesquisadora principal do estudo Solidarity (não remunerado)	Painelista
Patrícia Parreira*	Não há conflitos de interesse	Revisão sistemática
Rachel Riera*	Não há conflitos de interesse	Metodologista da diretriz Revisão sistemática
Regis Rosa	Relacionados à diretriz: membro do comitê diretivo dos estudos COALIZÃO (não remunerado), avaliando hidroxicloroquina, azitromicina, corticoesteroides e tocilizumabe, financiado pelo Ministério da Saúde, EMS e hospitais filantrópicos.	Painelista
Sergio Cimerman	Relacionados à diretriz: fornecimento de medicação para pesquisa sobre nitazoxanida Não relacionados à diretriz: recebeu financiamento da Merck, AbbVie, United Medical, Janssen, Pfizer, Novartis, Dr Reddy's, Roche, Farmoquímica, Apsen, Amgen Não relacionados à diretriz: Sociedade Brasileira de Infectologia (SBI)	Painelista
Verônica Colpani*	Não há conflitos de interesse	Metodologista da diretriz Revisão sistemática

* Participantes não possuíam poder de voto.

Participação nos painéis de recomendações

**Table:**

Participante*	Painel de 5 maio de 2020	Painel de 8 de maio de 2020	Painel de 13 de maio de 2020
Alexandre Zavascki	X	X	X
Amilton Silva Júnior	-	X	X
Angela Bagattini	-	-	X
Bruno Tavares	X	X	X
Cassia Pagano	X	-	X
Cinara Stein	X	X	X
Clovis Cunha	X	X	X
Daniela Pachito	-	-	-
Débora Graf	X	-	X
Felipe Dal Pizzol	X	-	X
Flávia Medeiros	-	-	X
Gabriela Brito	-	-	-
Haliton Oliveira Júnior	X	X	X
Jessica Matuoka	-	-	X
José Chatkin	-	-	-
Lays Pires Marra	-	-	-
Leandro Fritscher	X	X	X
Luciano Azevedo	X	-	-
Maicon Falavigna	X	X	X
Marcelo Gazzana	X	X	X
Michele Nunes	X	X	X
Mirian Dalben	X	X	
Patrícia Parreira	-	-	X
Rachel Riera	X	X	X
Regis Rosa	X	X	X
Sergio Cimerman	X	X	X
Verônica Colpani	X	X	X

* Todos os participantes revisaram e contribuíram com a redação da diretriz e aprovaram o documento final. A reunião do dia 5 de maio de 2020 teve duração de 3 horas e incluiu a discussão das questões sobre hidroxicloroquina/cloroquina, tocilizumabe e ritonavir/lopinavir e dos critérios da gravidade da doença. A reunião do dia 8 de maio de 2020 teve duração de 2 horas e meia e incluiu a discussão das questões sobre glicocorticoides, hidroxicloroquina/cloroquina associada à azitromicina, oseltamivir e antibioticoterapia. A reunião do dia 13 de maio de 2020 teve duração de 1 hora e meia e incluiu a discussão da questão sobre heparina e redação do texto.

## Apêndice 2 - Estratégias de busca das revisões sistemática

Pergunta 1 - Cloroquina e/ou hidroxicloroquina comparadas a tratamento convencional em paciente com infecção por COVID-19

Data da busca: 24 de abril de 2020

**Table:**

Base de dados	Estratégia de busca
Cochrane Library	#1 MeSH descriptor: [Coronavirus] explode all trees
	#2 "COVID-19" OR (COVID) OR (Coronavirus) OR (SARS-CoV-2) OR (Coronaviruses) OR (Deltacoronavirus) OR (Deltacoronaviruses) OR "Munia coronavirus HKU13" OR (Coronavirus HKU15) OR (Coronavirus, Rabbit) OR (Rabbit Coronavirus) OR (Coronaviruses, Rabbit) OR (Rabbit Coronaviruses) OR "Bulbul coronavirus HKU11" OR "Thrush coronavirus HKU12"
	#3 #1 OR #2
	#4 MeSH descriptor: [Hydroxychloroquine] explode all trees
	#5 MeSH descriptor: [Chloroquine] explode all trees
	#6 MeSH descriptor: [Antimalarials] explode all trees
	#7 (Hydroxychloroquine) OR (Oxychlorochin) OR (Oxychloroquine) OR (Hydroxychlorochin) OR (Plaquenil) OR (Hydroxychloroquine Sulfate) OR "HydroxychloroquineSulfate (1:1) Salt" OR (Hidroxicloroquina) OR (Hydroxychloroquine) OR (Hydroxychloroquinum) OR (Oxichlorochine) OR (Oxichloroquine) OR Chlorochin OR CloroquinaOR Cloroquine OR Chloroquine OR (Antimalarials) OR (Antimalarial Agents) OR (Agents, Antimalarial) OR (Antimalarial Drugs) OR (Drugs, Antimalarial) OR (Anti-Malarials) OR (Anti Malarials) OR "(N4-(7-Chloro-4-quinolinyl)-N1,N1-diethyl-1,4-pentanediamine)" OR Hydroquin OR Axemal OR Dolquine OR Quensyl OR Quinoric OR Plaquenil
	#8 #4 OR #5 OR #6 OR #7
	#9 #3 AND #8
Embase	#1 'coronavirinae' OR 'coronavirinae'/exp OR coronavirinae OR 'corona virus'/exp OR 'corona virus' OR 'coronavirus'/exp OR coronavirus OR 'covid-19' OR covid OR 'sars-cov-2' OR coronaviruses OR deltacoronavirus OR deltacoronaviruses OR 'munia coronavirus hku13' OR 'coronavirus hku15' OR 'coronavirus, rabbit' OR 'rabbit coronavirus' OR 'coronaviruses, rabbit' OR 'rabbit coronaviruses' OR 'bulbul coronavirus hku11' OR 'thrush coronavirus hku12'
	#2 'hydroxychloroquine' OR 'hydroxychloroquine'/exp OR hydroxychloroquine OR '7 chloro 4 [4 [ethyl (2 hydroxyethyl) amino] 1 methylbutylamino] quinoline'/exp OR '7 chloro 4 [4 [ethyl (2 hydroxyethyl) amino] 1 methylbutylamino] quinoline' OR '7 chloro 4 [4 [ethyl (2 hydroxyethyl) amino] 1 methylbutylamino] quinoline diphosphate'/exp OR '7 chloro 4 [4 [ethyl (2 hydroxyethyl) amino] 1 methylbutylamino] quinoline diphosphate' OR 'chloroquinol'/exp OR chloroquinol OR 'ercoquin'/exp OR ercoquin OR 'hydrochloroquine'/exp OR hydrochloroquine OR 'hydrocloroquine'/exp OR hydrocloroquine OR 'oxychloroquine'/exp OR oxychloroquine OR 'quensyl'/exp OR quensyl OR 'sn 8137'/exp OR 'sn 8137' OR oxychlorochin OR hydroxychlorochin OR plaquenil OR 'hydroxychloroquine sulfate' OR 'hydroxychloroquine sulfate (1:1) salt' OR hidroxicloroquina OR hydroxychloroquinum OR oxichlorochine OR oxichloroquine OR 'chloroquine' OR 'chloroquine'/exp OR chloroquine OR '4 (4 diethylamino 1 methylbutylamino) 7 chlorchinolin diphosphate' OR '4 (4 diethylamino 1 methylbutylamino) 7 chlorchinolin sulfate' OR '4 (4 diethylamino 1 methylbutylamino) 7 chlorchinolin sulfate' OR '4 (4 diethylamino 1 methylbutylamino) 7 chloroquinoline' OR '7 chloro 4 (4 diethylamino 1 methylbutylamino) quinoline' OR '7 chloro 4 (4 diethylamino 1 methylbutylamino) quinoline diphosphate' OR '7 chloro 4 (4 diethylamino 1 methylbutylamino) quinoline' OR 'a-cq' OR amokin OR amokine OR anoclor OR aralan OR aralen OR 'aralen hydrochloride' OR 'aralen phosphate' OR aralene OR arechin OR arechine OR arequine OR arthrochin OR arthrochine OR arthroquine OR artrichin OR artrichine OR artriquine OR avloclor OR avoclor OR bemaphata OR bemaphate OR bemasulph OR bipiquin OR cadiquin OR chemochin OR chemochine OR chingamine OR chingaminum OR chloraquine OR chlorochin OR chlorochine OR chlorofoz OR chloroquin OR 'chloroquin phosphate' OR 'chloroquine diphosphate' OR 'chloroquine disulfate' OR 'chloroquine disulphate' OR 'chloroquine hydrochloride' OR 'chloroquine phosphate' OR 'chloroquine streuli' OR 'chloroquine sulfate' OR 'chloroquine sulphate' OR chloroquinesulphate OR 'chloroquini diphosphas' OR 'chloroquinum diphosphoricum' OR chlorquin OR chlorquine OR choloquine OR 'choroquine sulfate' OR 'choroquine sulphate' OR cidanchin OR 'clo-kit junior' OR clorichina OR clorichine OR cloriquine OR clorochina OR delagil OR delagyl OR dichinalex OR diclokin OR diquinalex OR diroquine OR emquin OR genocin OR gontochin OR gontochine OR gontoquine OR heliopar OR imagon OR iroquine OR klorokin OR klorokine OR klorokinfosfat OR lagaquin OR malaquin OR malarex OR malarivon OR malaviron OR maliaquine OR maquine OR mesylith OR mexaquin OR mirquin OR nivachine OR nivaquin OR nivaquine OR 'nivaquine (b)' OR 'nivaquine b' OR 'nivaquine dp' OR 'nivaquine forte' OR 'p roquine' OR quinachlor OR quingamine OR repal OR resochen OR resochene OR resochin OR 'resochin junior' OR resochina OR resochine OR resochinon OR resoquina OR resoquine OR reumachlor OR roquine OR 'rp 3377' OR rp3377 OR sanoquin OR sanoquine OR silbesan OR siragan OR sirajan OR 'sn 7618' OR sn7618 OR solprina OR solprine OR tresochin OR tresochine OR tresoquine OR trochin OR trochine OR troquine OR 'w 7618' OR w7618 OR 'win 244' OR win244 OR 'antimalarial agent'/exp OR 'antimalarial agent' OR 'anti malaria drug'/exp OR 'anti malaria drug' OR 'antimalaria agent'/exp OR 'antimalaria agent' OR 'antimalaria drug'/exp OR 'antimalaria drug' OR 'antimalaria drug, synthetic'/exp OR 'antimalaria drug, synthetic' OR 'antimalarial'/exp OR antimalarial OR 'antimalarial drug'/exp OR 'antimalarial drug' OR 'antimalarials'/exp OR antimalarials OR 'antipaludean agent'/exp OR 'antipaludean agent' OR 'antiplasmodic agent'/exp OR 'antiplasmodic agent' OR 'synthetic antimalaria agent'/exp OR 'synthetic antimalaria agent'
	#3 #1 AND #2
	#4 #3 AND [embase]/lim NOT ([embase]/lim AND [medline]/lim)
LILACS	#1 MH:"Coronavirus" OR MH: B04.820.504.540.150$ OR (Coronavirus) OR "COVID-19" OR (COVID) OR (SARS-CoV-2) OR (Deltacoronavirus) OR (Coronaviruses)
	#2 MH:"Hydroxychloroquine" OR MH:"Hidroxicloroquina" OR MH:D03.633.100.810.050.180.350$ OR (Hydroxychloroquine) OR (Hidroxicloroquina) OR (Hydroxychlorochin) OR (Hydroxychloroquine Sulfate) OR "Hydroxychloroquine Sulfate (1:1) Salt" OR (Oxychlorochin) OR (Oxychloroquine) OR (Plaquenil) OR (Oxicloroquina) OR MH:"Cloroquina" OR MH:"Chloroquine" OR MH:D03.633.100.810.050.180$ OR (Cloroquina) OR (Chloroquine) OR (Aralen) OR (Arechine) OR (Arequin) OR (Chingamin) OR (Chlorochin) OR (Chloroquine Sulfate) OR (Chloroquine Sulphate) OR (Khingamin) OR (Nivaquine) OR (Sulfate, Chloroquine) OR (Sulphate, Chloroquine) OR MH:"Antimaláricos" OR MH:"Antimalarials" OR MH:D27.505.954.122.250.100.085$ OR (Antimaláricos) OR (Antimalarials) OR (Agents, Antimalarial) OR (Anti Malarials) OR (Anti-Malarials) OR (Antimalarial Agents) OR (Antimalarial Drugs) OR (Drugs, Antimalarial)
	#3 #1 AND #2
	#4 in [Lilacs]
MEDLINE	#1 "Coronavirus"[Mesh] OR "COVID-19" OR (COVID) OR (Coronavirus) OR (SARS-CoV-2) OR (Coronaviruses) OR (Deltacoronavirus) OR (Deltacoronaviruses) OR "Munia coronavirus HKU13" OR (Coronavirus HKU15) OR (Coronavirus, Rabbit) OR (Rabbit Coronavirus) OR (Coronaviruses, Rabbit) OR (Rabbit Coronaviruses) OR "Bulbul coronavirus HKU11" OR "Thrush coronavirus HKU12"
(via PubMed^®^)	#2 "Hydroxychloroquine"[Mesh] OR (Hydroxychloroquine) OR (Oxychlorochin) OR (Oxychloroquine) OR (Hydroxychlorochin) OR (Plaquenil) OR (Hydroxychloroquine Sulfate) OR "Hydroxychloroquine Sulfate (1:1) Salt" OR (Hidroxicloroquina) OR (Hydroxychloroquine) OR (Hydroxychloroquinum) OR (Oxichlorochine) OR (Oxichloroquine) OR "Chloroquine"[Mesh] OR Chlorochin OR Cloroquina OR Cloroquine OR Chloroquine OR "Antimalarials"[Mesh] OR (Antimalarials) OR (Antimalarial Agents) OR
	(Agents, Antimalarial) OR (Antimalarial Drugs) OR
	(Drugs, Antimalarial) OR (Anti-Malarials) OR
	(Anti Malarials) OR "(N4-(7-Chloro-4-quinolinyl) -N1, N1-diethyl-1,4-pentanediamine)" OR Hydroquin OR Axemal OR Dolquine OR Quensyl OR Quinoric
	#3 #1 AND #2
OpenGrey	#1 "COVID-19" OR (COVID) OR (Coronavirus) OR (SARS-CoV-2) OR (Coronaviruses) OR (Deltacoronavirus) OR (Deltacoronaviruses)
ClinicalTrials.gov	#1 "COVID-19" OR (COVID) OR (Coronavirus) OR (SARS-CoV-2) OR (Coronaviruses) OR (Deltacoronavirus) OR (Deltacoronaviruses)
	#2 Hydroxychloroquine OR Oxychlorochin OR Oxychloroquine OR Hydroxychlorochin OR Plaquenil OR Chlorochin OR Cloroquina OR Cloroquine OR chloroquine OR Antimalarials OR Antimalarial
	#3 #1 AND #2
WHO-ICTRP*	#1 "COVID-19" OR (COVID) OR (Coronavirus) OR (SARS-CoV-2) OR (Coronaviruses) OR (Deltacoronavirus) OR (Deltacoronaviruses)
	#2 Hydroxychloroquine OR Oxychlorochin OR Oxychloroquine OR Hydroxychlorochin OR Plaquenil OR Chlorochin OR Cloroquina OR Cloroquine OR chloroquine OR Antimalarials OR Antimalarial
	#3 #1 AND #2

* A busca foi realizada em 19 de março de 2020 e não pôde ser atualizada pelo fato de a base estar indisponível.

**Pergunta 2 -** Hidroxicloroquina/cloroquina associada à azitromicina comparada a não utilizar em paciente com infecção por COVID-19

Data da busca: 28 de abril de 2020

**Table:**

Base de dados	Estratégia de busca
MEDLINE (via PubMed®)	#1 "Coronavirus"[Mesh] OR "Covid-19" OR (COVID) OR (Coronavirus) OR (SARS-CoV-2) OR (Coronaviruses) OR (Deltacoronavirus) OR (Deltacoronaviruses) OR "Munia coronavirus HKU13" OR (Coronavirus HKU15) OR (Coronavirus, Rabbit) OR (Rabbit Coronavirus) OR (Coronaviruses, Rabbit) OR (Rabbit Coronaviruses) OR "Bulbul coronavirus HKU11" OR "Thrush coronavirus HKU12" OR "novel coronavirus" OR "covid-19" OR "sarscov 2" OR "Betacoronavirus*"
	#2 "Anti-Bacterial Agents" [mesh] OR "Anti-Bacterial Agents" OR "Agents, Anti-Bacterial" OR "Anti-Bacterial Agents" OR "Antibacterial Agents" OR "Agents, Antibacterial" OR "Anti-Bacterial Compounds" OR "Anti-Bacterial Compounds" OR "Compounds, Anti-Bacterial" OR "Bacteriocidal Agents" OR "Agents, Bacteriocidal" OR "Bacteriocides" OR "Anti-Mycobacterial Agents" OR "Agents, Anti-Mycobacterial" OR "Anti Mycobacterial Agents" OR "Antimycobacterial Agents" OR "Agents, Antimycobacterial" OR "Antibiotics" OR "Antibiotic" OR "antimicrobials" OR "antibacterials" OR "Azithromycin" [mesh] OR "Azythromycin" OR "Sumamed" OR "Toraseptol" OR "Vinzam" OR "CP-62993" OR "CP 62993" OR "CP62993" OR "Zithromax" OR "Azitrocin" OR "Azadose" OR "Ultreon" OR "Zitromax" OR "Azithromycin Dihydrate" OR "Dihydrate, Azithromycin" OR "Azithromycin Monohydrate" OR "Monohydrate, Azithromycin" OR "Goxal" OR "Zentavion"
	#3 "Hydroxychloroquine"[Mesh] OR (Hydroxychloroquine) OR (Oxychlorochin) OR (Oxychloroquine) OR (Hydroxychlorochin) OR (Plaquenil) OR (Hydroxychloroquine Sulfate) OR "Hydroxychloroquine Sulfate (1:1) Salt" OR (Hidroxicloroquina) OR (Hydroxychloroquine) OR (Hydroxychloroquinum) OR (Oxichlorochine) OR (Oxichloroquine) OR "Chloroquine"[Mesh] OR Chlorochin OR Cloroquina OR Cloroquine OR Chloroquine OR "Antimalarials"[Mesh] OR (Antimalarials) OR (Antimalarial Agents) OR (Agents, Antimalarial) OR (Antimalarial Drugs) OR (Drugs, Antimalarial) OR (Anti-Malarials) OR (Anti Malarials) OR "(N4-(7-Chloro-4-quinolinyl)-N1,N1-diethyl-1,4- pentanediamine)" OR Hydroquin OR Axemal OR Dolquine OR Quensyl OR Quinoric
	#4 #1 AND #2 AND #3
Embase	#1 ('sars-related coronavirus'/exp OR 'sars-related coronavirus' OR 'covid 19' OR 'Covid-19' OR 'novel coronavirus' OR 'sars-cov2' OR 'sars-ncov' OR 'sars-cov-2') AND [embase]/lim
	#2 'antibiotic agent'/exp OR 'azithromycin'/exp OR 'Antibiotics' OR 'Antibiotic'
	#3 'hydroxychloroquine' OR 'hydroxychloroquine'/exp OR hydroxychloroquine OR '7 chloro 4 [4 [ethyl (2 hydroxyethyl) amino] 1 methylbutylamino] quinoline'/exp OR '7 chloro 4 [4 [ethyl (2 hydroxyethyl) amino] 1 methylbutylamino] quinoline' OR '7 chloro 4 [4 [ethyl (2 hydroxyethyl) amino] 1 methylbutylamino] quinoline diphosphate'/exp OR '7 chloro 4 [4 [ethyl (2 hydroxyethyl) amino] 1 methylbutylamino] quinoline diphosphate' OR 'chloroquinol'/exp OR chloroquinol OR 'ercoquin'/exp OR ercoquin OR 'hydrochloroquine'/exp OR hydrochloroquine OR 'hydrocloroquine'/exp OR hydrocloroquine OR 'oxychloroquine'/exp OR oxychloroquine OR 'quensyl'/exp OR quensyl OR 'sn 8137'/exp OR 'sn 8137' OR oxychlorochin OR hydroxychlorochin OR plaquenil OR 'hydroxychloroquine sulfate' OR 'hydroxychloroquine sulfate (1:1) salt' OR hidroxicloroquina OR hydroxychloroquinum OR oxichlorochine OR oxichloroquine OR 'chloroquine' OR 'chloroquine'/exp OR chloroquine OR '4 (4 diethylamino 1 methylbutylamino) 7 chlorchinolin diphosphate' OR '4 (4 diethylamino 1 methylbutylamino) 7 chlorchinolin sulfate' OR '4 (4 diethylamino 1 methylbutylamino) 7 chlorchinolin sulfate' OR '4 (4 diethylamino 1 methylbutylamino) 7 chloroquinoline' OR '7 chloro 4 (4 diethylamino 1 methylbutylamino) quinoline' OR '7 chloro 4 (4 diethylamino 1 methylbutylamino) quinoline diphosphate' OR '7 chloro 4 (4 diethylamino 1 methylbutylamino) quinoline' OR 'a-cq' OR amokin OR amokine OR anoclor OR aralan OR aralen OR 'aralen hydrochloride' OR 'aralen phosphate' OR aralene OR arechin OR arechine OR arequine OR arthrochin OR arthrochine OR arthroquine OR artrichin OR artrichine OR artriquine OR avloclor OR avoclor OR bemaphata OR bemaphate OR bemasulph OR bipiquin OR cadiquin OR chemochin OR chemochine OR chingamine OR chingaminum OR chloraquine OR chlorochin OR chlorochine OR chlorofoz OR chloroquin OR 'chloroquin phosphate' OR 'chloroquine diphosphate' OR 'chloroquine disulfate' OR 'chloroquine disulphate' OR 'chloroquine hydrochloride' OR 'chloroquine phosphate' OR 'chloroquine streuli' OR 'chloroquine sulfate' OR 'chloroquine sulphate' OR chloroquinesulphate OR 'chloroquini diphosphas' OR 'chloroquinum diphosphoricum' OR chlorquin OR chlorquine OR choloquine OR 'choroquine sulfate' OR 'choroquine sulphate' OR cidanchin OR 'clo-kit junior' OR clorichina OR clorichine OR cloriquine OR clorochina OR delagil OR delagyl OR dichinalex OR diclokin OR diquinalex OR diroquine OR emquin OR genocin OR gontochin OR gontochine OR gontoquine OR heliopar OR imagon OR iroquine OR klorokin OR klorokine OR klorokinfosfat OR lagaquin OR malaquin OR malarex OR malarivon OR malaviron OR maliaquine OR maquine OR mesylith OR mexaquin OR mirquin OR nivachine OR nivaquin OR nivaquine OR 'nivaquine (b)' OR 'nivaquine b' OR 'nivaquine dp' OR 'nivaquine forte' OR 'p roquine' OR quinachlor OR quingamine OR repal OR resochen OR resochene OR resochin OR 'resochin junior' OR resochina OR resochine OR resochinon OR resoquina OR resoquine OR reumachlor OR roquine OR 'rp 3377' OR rp3377 OR sanoquin OR sanoquine OR silbesan OR siragan OR sirajan OR 'sn 7618' OR sn7618 OR solprina OR solprine OR tresochin OR tresochine OR tresoquine OR trochin OR trochine OR troquine OR 'w 7618' OR w7618 OR 'win 244' OR win244 OR 'antimalarial agent'/exp OR 'antimalarial agent' OR 'anti malaria drug'/exp OR 'anti malaria drug' OR 'antimalaria agent'/exp OR 'antimalaria agent' OR 'antimalaria drug'/exp OR 'antimalaria drug' OR 'antimalaria drug, synthetic'/exp OR 'antimalaria drug, synthetic' OR 'antimalarial'/exp OR antimalarial OR 'antimalarial drug'/exp OR 'antimalarial drug' OR 'antimalarials'/exp OR antimalarials OR 'antipaludean agent'/exp OR 'antipaludean agent' OR 'antiplasmodic agent'/exp OR 'antiplasmodic agent' OR 'synthetic antimalaria agent'/exp OR 'synthetic antimalaria agent'
	#3 #1 AND #3 AND #4
Cochrane Library	# 1 MeSH descriptor: [SARS Virus] explode all trees
	#2 MeSH descriptor: [Coronavirus] explode all trees
	#3 "Covid-19" OR (Covid) OR (Coronavirus) OR (SARS-CoV-2) OR (Coronaviruses) OR (Deltacoronavirus) OR (Deltacoronaviruses) OR "Munia coronavirus HKU13" OR (Coronavirus HKU15) OR (Coronavirus, Rabbit) OR (Rabbit Coronavirus) OR (Coronaviruses, Rabbit) OR (Rabbit Coronaviruses) OR "Bulbul coronavirus HKU11" OR "Thrush coronavirus HKU12"
	#4 #1 OR #2 OR #3
	#5 MeSH descriptor: [Anti-Bacterial Agents] explode all trees
	#6 MeSH descriptor: [Azithromycin] explode all trees
	#7 #5 OR #6
	#8 MeSH descriptor: [Hydroxychloroquine] explode all trees
	#9 MeSH descriptor: [Chloroquine] explode all trees
	#10 MeSH descriptor: [Antimalarials] explode all trees
	#11 (Hydroxychloroquine) OR (Oxychlorochin) OR (Oxychloroquine) OR (Hydroxychlorochin) OR (Plaquenil) OR (Hydroxychloroquine Sulfate) OR "Hydroxychloroquine Sulfate (1:1) Salt" OR (Hidroxicloroquina) OR (Hydroxychloroquine) OR (Hydroxychloroquinum) OR (Oxichlorochine) OR (Oxichloroquine) OR Chlorochin OR Cloroquina OR Cloroquine OR Chloroquine OR (Antimalarials) OR (Antimalarial Agents) OR (Agents, Antimalarial) OR (Antimalarial Drugs) OR (Drugs, Antimalarial) OR (Anti-Malarials) OR (Anti Malarials) OR "(N4-(7-Chloro-4-quinolinyl)-N1,N1-diethyl1,4-pentanediamine)" OR Hydroquin OR Axemal OR Dolquine OR Quensyl OR Quinoric OR Plaquenil
	#12 #8 OR #9 OR #10 OR #11
	#4 AND #7 AND #12
meDrxiv	("Anti-Bacterial Agents" OR antibiotics OR Azithromycin) AND (Hydroxychloroquine OR Oxychlorochin OR Oxychloroquine OR Hydroxychlorochin OR Plaquenil OR Chlorochin OR Cloroquina OR Cloroquine OR chloroquine OR Antimalarials OR Antimalarial)
OpenGrey	("Anti-Bacterial Agents" OR antibiotics OR Azithromycin) AND (Hydroxychloroquine OR Oxychlorochin OR Oxychloroquine OR Hydroxychlorochin OR Plaquenil OR Chlorochin OR Cloroquina OR Cloroquine OR chloroquine OR Antimalarials OR Antimalarial)
ClinicalTrials.gov	Covid 19 OR Covid-19 OR SARS-CoV 2 OR SARS-CoV-2 OR nCoV 2019 OR severe acute respiratory syndrome coronavirus 2 / ("Anti-Bacterial Agents" OR antibiotics OR Azithromycin) AND (Hydroxychloroquine OR Oxychlorochin OR Oxychloroquine OR Hydroxychlorochin OR Plaquenil OR Chlorochin OR Cloroquina OR Cloroquine OR chloroquine OR Antimalarials OR Antimalarial)

**Pergunta 3 -** Oseltamivir comparado a não utilizar em paciente com infecção por COVID-19

Data da busca: 27 de abril de 2020

Mesma estratégia de busca da pergunta 4.

**Pergunta 4 -** Lopinavir associado a ritonavir comparado a cuidados padrão em paciente com infecção por COVID-19

Data da busca: 27 de abril de 2020

**Table:**

Base de dados	Estratégia de busca
Medline (via Pubmed®)	#1 (("Anti-Retroviral Agents"[Mesh] OR anti-retroviral agent OR antiretroviral agent OR "Anti-Retroviral Agents" [Pharmacological Action] OR "CCR5 Receptor Antagonists"[Mesh] OR CCR5 Receptor Antagonists OR "HIV Fusion Inhibitors"[Mesh] OR fusion inhibitor OR "HIV Integrase Inhibitors"[Mesh] OR Integrase Inhibitors OR "HIV Protease Inhibitors"[Mesh] OR Protease Inhibitors OR "Lopinavir"[Mesh] OR "lopinavir-ritonavir drug combination" [Supplementary Concept] OR "Ritonavir"[Mesh] OR "favipiravir" [Supplementary Concept] OR lopinavir OR ritonavir OR favipiravir OR "Antiviral Agents"[Mesh] OR "Oseltamivir"[Mesh] OR antiviral OR antiviral agent OR oseltamivir))
	#2 ((novel coronavirus OR covid-19 OR covid 19 OR covid - 19 OR sars-cov-2 OR sarscov 2))
	#3 #1 AND #2
Embase	#1 ('sars-related coronavirus'/exp OR 'sars-related coronavirus' OR 'covid 19' OR 'covid-19' OR 'novel coronavirus' OR 'sars-cov2' OR 'sars-ncov' OR 'sars-cov-2') AND [embase]/lim
	#2 ('antiretrovirus agent'/exp OR 'antiretrovirus agent' OR 'antiretoviral agent' OR 'anti-retroviral agent'/exp OR 'anti-retroviral agent' OR 'chemokine receptor ccr5 antagonist'/exp OR 'chemokine receptor ccr5 antagonist' OR 'ccr5 receptor antagonists'/exp OR 'ccr5 receptor antagonists' OR 'human immunodeficiency virus fusion inhibitor'/exp OR 'human immunodeficiency virus fusion inhibitor' OR 'hiv fusion inhibitors'/exp OR 'hiv fusion inhibitors' OR 'hiv integrase inhibitors'/exp OR 'hiv integrase inhibitors' OR 'integrase inhibitor'/exp OR 'integrase inhibitor' OR 'hiv protease inhibitors'/exp OR 'hiv protease inhibitors' OR 'human immunodeficiency virus proteinase inhibitor'/exp OR 'human immunodeficiency virus proteinase inhibitor' OR 'lopinavir'/exp OR 'lopinavir' OR 'lopinavir plus ritonavir'/exp OR 'lopinavir plus ritonavir' OR 'ritonavir'/exp OR 'ritonavir' OR 'favipiravir'/exp OR 'favipiravir' OR 'antivirus agent'/exp OR 'antiviral' OR 'oseltamivir'/exp OR 'oseltamivir' OR 'antiviral agent' OR 'antivirus agent') AND [embase]/lim
	#3 #1 AND #2
Cochrane Library	#1 severe acute respiratory syndrome coronavirus OR severe acute respiratory syndrome coronavirus 2 OR SARS-CoV-2 OR SARS CoV 2
	OR SARS-CoV 2 OR coronavirus disease 2019 OR COVID 19 OR COVID 19 OR nCoV 2019 in All Text
	#2 (antiretroviral agent OR Anti Retroviral Agents OR HIV Fusion Inhibitors OR fusion inhibitor OR HIV Integrase Inhibitors OR HIV Protease Inhibitors OR Protease Inhibitors OR Lopinavir OR lopinavir-ritonavir drug combination OR lopinavir ritonavir OR Ritonavir OR favipiravir OR Oseltamivir) in All Text - (Word variations have been searched)
	#3 #1 AND #2
meDrxiv	#1 (SARS CoV 2 OR COVID-19)
	#2 (antiretroviral agente OR Anti-Retroviral Agents OR HIV Fusion Inhibitors OR Protease Inhibitors)
	#3 #1 AND #2
OpenGrey	#1 (covid-19 OR SARS-CoV2 OR severe acute respiratory syndrome coronavirus)
	#2 (antiretroviral agents OR Anti Retroviral Agents OR HIV Fusion Inhibitors OR fusion inhibitor OR HIV Integrase Inhibitors OR HIV Protease Inhibitors OR Protease Inhibitors)
	#3 #1 AND #2
ClinicalTrials.gov	covid-19 OR sars-cov-2 OR sars-cov OR novel coronavirus

**Pergunta 5 -** Corticoides comparados a não utilizar em paciente com infecção por COVID-19

Data da busca: 27 de abril de 2020

**Table:**

Base de dados	Estratégia de busca
Medline (via Pubmed®)	#1 ((((novel coronavirus OR covid-19 OR covid 19 OR covid - 19 OR sars-cov-2 OR sarscov 2))))
	#2 ("Adrenal Cortex Hormones"[Mesh] OR "Hydrocortisone"[Mesh] OR "Prednisone"[Mesh] OR "Prednisolone"[Mesh] OR "Beclomethasone"[Mesh] OR "Fluticasone"[Mesh] OR "Budesonide"[Mesh] OR corticosteroids OR "Methylprednisolone"[Mesh] OR methylprednisolone OR corticoids OR cortisol OR Hydrocortisone OR prednisone OR prednisolone OR beclomethasone OR fluticasone OR budesonide)
	#3 #1 AND #2
Embase	#1 ('corticosteroid'/exp OR 'corticosteroid' OR 'hydrocortisone'/exp OR 'hydrocortisone' OR 'prednisone'/exp OR 'prednisone' OR 'prednisolone'/exp OR 'prednisolone' OR 'beclomethasone'/exp OR 'beclomethasone' OR 'fluticasone'/exp OR 'fluticasone' OR 'budesonide'/exp OR 'budesonide' OR 'corticosteroids'/exp OR 'corticosteroids' OR 'methylprednisolone'/exp OR 'methylprednisolone' OR 'corticoids' OR 'cortisol'/exp OR 'cortisol') AND [embase]/lim
	#2 'sars-related coronavirus'/exp OR 'sars-related coronavirus' OR 'covid 19' OR 'covid-19' OR 'novel coronavirus' OR 'sars-cov2' OR 'sars-ncov' OR 'sars-cov-2'
	#3 #1 AND #2
Cochrane Library	#1 MeSH descriptor: [Coronavirus] explode all trees
	#2 "COVID-19" OR (COVID) OR (Coronavirus) OR (SARS-CoV-2) OR (Coronaviruses) OR (Deltacoronavirus) OR (Deltacoronaviruses) OR "Munia coronavirus HKU13" OR (Coronavirus HKU15) OR (Coronavirus, Rabbit) OR (Rabbit Coronavirus) OR (Coronaviruses, Rabbit) OR (Rabbit Coronaviruses) OR "Bulbul coronavirus HKU11" OR "Thrush coronavirus HKU12" OR "SARS CoV"
	#3 MeSH descriptor: [SARS Virus] explode all trees
	#4 #1 OR #2 OR #3
	#5 MeSH descriptor: [Glucocorticoids] explode all trees OR corticosteroid OR Glucocorticoids OR hydrocortisone OR MeSH descriptor: [Hydroxycorticosteroids] explode all trees OR Hydroxycorticosteroids OR MeSH descriptor: [Prednisone] explode all trees OR MeSH descriptor: [Beclomethasone] explode all trees OR Beclomethasone OR MeSH descriptor: [Fluticasone] explode all trees OR Fluticasone OR MeSH descriptor: [Budesonide] explode all trees OR Budesonide OR MeSH Descriptor:[Methylprednisolone] explode all trees OR Methylprednisolone OR MeSH descriptor: [Hydrocortisone] explode all trees OR cortisol OR Hydrocortisone
	#6 #4 AND #5
meDrxiv	#1("SARS CoV" OR "SARS CoV 2" OR "COVID-19")
	#2 (corticosteroid OR hydrocortisone OR prednisone OR beclomethasone OR budesonide OR fluticasone OR methylprednisolone OR cortisol)
	#3 #1 AND #2
OpenGrey	#1 (corticosteroid OR hydrocortisone OR prednisone OR beclomethasone OR budesonide OR fluticasone OR methylprednisolone OR cortisol)
	#2. (covid-19 OR SARS-CoV2 OR SARS-CoV OR severe acute respiratory syndrome coronavirus)
	#3 #1 AND #2
ClinicalTrials.gov	corticosteroid OR hydrocortisone OR prednisone OR beclomethasone OR budesonide OR fluticasone OR methylprednisolone OR cortisol | COVID 19 OR COVID-19 OR SARS-CoV 2 OR SARS-CoV-2 OR nCoV 2019 OR severe acute respiratory syndrome coronavirus 2

**Pergunta 6 -** Tocilizumabe comparado a não utilizar em paciente com infecção por COVID-19

Data da busca: 22 de abril de 2020

**Table:**

Base de dados	Estratégia de busca
Medline	((Coronavirus disease 2019 OR covid-19 OR covid 19 OR nCoV-2019 OR Severe acute respiratory syndrome coronavirus OR SARS-Cov-2 OR SARS-CoV2)) AND ("tocilizumab" [Supplementary Concept] OR tocilizumab OR tocilizumabe)
Embase	('coronavirus disease 2019'/exp OR 'coronavirus disease 2019' OR 'covid-19' OR 'covid 19'/exp OR 'covid 19' OR 'ncov-2019' OR 'severe acute respiratory syndrome coronavirus'/exp OR 'severe acute respiratory syndrome coronavirus' OR 'sars-cov-2' OR 'sars-cov2') AND [embase]/lim
	AND
	('tocilizumab'/exp OR 'tocilizumab') AND [embase]/lim
ClinicalTrials	tocilizumab | COVID-19 OR SARS-CoV-2 OR nov-2019 OR COVID 19

**Pergunta 7 -** Heparinas comparadas a não utilizar em paciente com infecção por COVID-19

Data da busca: 30 de abril de 2020

**Table:**

Base de dados	Estratégia de busca
Cochrane Library	#1 MeSH descriptor: [Coronavirus] explode all trees
	#2 "COVID-19" OR (COVID) OR (Coronavirus) OR (SARS-CoV-2) OR (Coronaviruses) OR (Deltacoronavirus) OR (Deltacoronaviruses) OR "Munia coronavirus HKU13" OR (Coronavirus HKU15) OR (Coronavirus, Rabbit) OR (Rabbit Coronavirus) OR (Coronaviruses, Rabbit) OR (Rabbit Coronaviruses) OR "Bulbul coronavirus HKU11" OR "Thrush coronavirus HKU12"
	#3 #1 OR #2
	#4 Review or Trials
Embase	#1 'coronaviridae'/exp OR 'coronavirus' OR 'coronaviridae' OR 'covid-19' OR covid OR coronavirus OR 'sars cov 2' OR coronaviruses OR deltacoronavirus OR deltacoronaviruses OR 'munia coronavirus hku13' OR (Coronavirus HKU15) OR (Coronavirus, Rabbit) OR (Rabbit Coronavirus) OR (Coronaviruses, Rabbit) OR (Rabbit Coronaviruses) OR 'bulbul coronavirus hku11' OR 'thrush coronavirus hku12'
	#2 'anticoagulant agent'/exp OR anticoagulants OR anticoagulant OR (Anticoagulation Agents) OR (Agents, Anticoagulation) OR (Anticoagulant Agents) OR (Agents, Anticoagulant) OR (Anticoagulant Drugs) OR (Drugs, Anticoagulant) OR (Indirect Thrombin Inhibitors) OR (Inhibitors, Indirect Thrombin) OR (Thrombin Inhibitors, Indirect)
	#3 'heparin'/exp OR heparin OR (Unfractionated Heparin) OR (Heparin, Unfractionated) OR (Heparinic Acid) OR Liquaemin OR (Sodium Heparin) OR (Heparin, Sodium) OR (Heparin Sodium) OR (alpha-Heparin) OR (alpha Heparin)
	#4 'low molecular weight heparin'/exp OR (Heparin, Low-Molecular-Weight) OR (Heparin, Low Molecular Weight) OR 'lmwh' OR (Low Molecular Weight Heparin) OR (Low-Molecular-Weight Heparin)
	#5 'enoxaparin'/exp OR enoxaparin OR enoxaparine OR 'pk-10,169' OR 'pk 10,169' OR 'pk10,169' OR 'pk-10169' OR 'pk 10169' OR 'pk10169' OR 'emt-967' OR 'emt 967' OR 'emt967' OR lovenox OR clexane OR 'emt-966' OR 'emt 966' OR 'emt966'
	#6 #2 OR #3 OR #4 OR #5
	#7 #1 AND #6
	#8 #7 AND [embase]/lim NOT ([embase]/lim AND [medline]/lim)
meDrxiv	"Heparin OR enoxaparin OR anticoagulant OR anticoagulants" (Abstract or Title)
MEDLINE (via PubMed®)	#1 "Coronavirus"[Mesh] OR "COVID-19" OR (COVID) OR (Coronavirus) OR (SARS-CoV-2) OR (Coronaviruses) OR (Deltacoronavirus) OR (Deltacoronaviruses) OR "Munia coronavirus HKU13" OR (Coronavirus HKU15) OR (Coronavirus, Rabbit) OR (Rabbit Coronavirus) OR (Coronaviruses, Rabbit) OR (Rabbit Coronaviruses) OR "Bulbul coronavirus HKU11" OR "Thrush coronavirus HKU12"
	#2 "Anticoagulants"[Mesh] OR Anticoagulants OR Anticoagulant OR (Anticoagulation Agents) OR (Agents, Anticoagulation) OR (Anticoagulant Agents) OR (Agents, Anticoagulant) OR (Anticoagulant Drugs) OR (Drugs, Anticoagulant) OR (Indirect Thrombin Inhibitors) OR (Inhibitors, Indirect Thrombin) OR (Thrombin Inhibitors, Indirect)
	#3 "Heparin"[Mesh] OR Heparin OR (Unfractionated Heparin) OR (Heparin, Unfractionated) OR (Heparinic Acid) OR Liquaemin OR (Sodium Heparin) OR (Heparin, Sodium) OR (Heparin Sodium) OR (alpha-Heparin) OR (alpha Heparin)
	#4 "Heparin, Low-Molecular-Weight"[Mesh] OR (Heparin, Low-Molecular-Weight) OR (Heparin, Low Molecular Weight) OR "LMWH" OR (Low Molecular Weight Heparin) OR (Low-Molecular-Weight Heparin)
	#5 "Enoxaparin"[Mesh] OR Enoxaparin OR Enoxaparine OR "PK-10,169" OR "PK 10,169" OR "PK10,169" OR "PK-10169" OR "PK 10169" OR "PK10169" OR "EMT-967" OR "EMT 967" OR "EMT967" OR Lovenox OR Clexane OR "EMT-966" OR "EMT 966" OR "EMT966"
	#6 "Nadroparin"[Mesh] OR Nadroparine OR "Nadroparin Calcium" OR "Calcium, Nadroparin" OR Fraxiparin OR Fraxiparine
	#7 "Dalteparin"[Mesh]" OR Tedelparin OR "Dalteparin Sodium" OR "Sodium, Dalteparin" OR Fragmin OR Fragmine
	#8 #2 OR #3 OR #4 OR #5 OR #6 OR #7
	#9 #1 AND #8
OpenGrey	#1 "COVID-19" OR (COVID) OR (Coronavirus) OR "SARS-CoV-2" OR (Coronaviruses) OR (Deltacoronavirus) OR (Deltacoronaviruses) OR "SARS CoV 2"
ClinicalTrials.gov	#1 "COVID-19" OR (COVID) OR (Coronavirus) OR (SARS-CoV-2) OR (Coronaviruses) OR (Deltacoronavirus) OR (Deltacoronaviruses)
	#2 (Anticoagulants) OR Heparin OR (Heparin, Low-Molecular-Weight) OR (Enoxaparin)
	#3 #1 AND #2
WHO Trial Registry Network COVID-19	#1 (Anticoagulants) OR (Anticoagulant)
	#2 Heparin
	#3 (Heparin, Low-Molecular-Weight) OR Enoxaparin
	#4 #3 OR #2 OR #1

**Pergunta 8 -** Antibioticoterapia comparada a não utilizar em paciente com COVID-19 sem evidência de infecção bacteriana

Data da busca: 27 de abril de 2020

**Table:**

Base de dados	Estratégia de busca
MEDLINE (via PubMed®)	#1 "Coronavirus"[Mesh] OR "Covid-19" OR (COVID) OR (Coronavirus) OR (SARS-CoV-2) OR (Coronaviruses) OR (Deltacoronavirus) OR (Deltacoronaviruses)OR "Munia coronavirus HKU13" OR (Coronavirus HKU15) OR (Coronavirus, Rabbit) OR (Rabbit Coronavirus) OR (Coronaviruses, Rabbit) OR (Rabbit Coronaviruses) OR "Bulbul coronavirus HKU11" OR "Thrush coronavirus HKU12" OR "novel coronavirus" OR "covid 19" OR "sarscov 2" OR "Betacoronavirus*"
	#2 "Anti-Bacterial Agents" [mesh] OR "Anti-Bacterial Agents" OR "Agents, Anti-Bacterial" OR "Anti-Bacterial Agents" OR "Antibacterial Agents" OR "Agents, Antibacterial" OR "Anti-Bacterial Compounds" OR "Anti-Bacterial Compounds" OR "Compounds, Anti-Bacterial" OR "Bacteriocidal Agents" OR "Agents, Bacteriocidal" OR "Bacteriocides" OR "Anti-Mycobacterial Agents" OR "Agents, Anti-Mycobacterial" OR "Anti Mycobacterial Agents" OR "Antimycobacterial Agents" OR "Agents, Antimycobacterial" OR "Antibiotics" OR "Antibiotic" OR "antimicrobials" OR "antibacterials" OR "Azithromycin" [mesh] OR "Azythromycin" OR "Sumamed" OR "Toraseptol" OR "Vinzam" OR "CP-62993" OR "CP 62993" OR "CP62993" OR "Zithromax" OR "Azitrocin" OR "Azadose" OR "Ultreon" OR "Zitromax" OR "Azithromycin Dihydrate" OR "Dihydrate, Azithromycin" OR "Azithromycin Monohydrate" OR "Monohydrate, Azithromycin" OR "Goxal" OR "Zentavion" OR "Vancomycin" [mesh] OR "Vancomycin" OR "Ceftriaxone" [mesh] OR "Ceftriaxone" OR "Cefepime" [mesh] OR "Cefepime" OR "Levofloxacin" [mesh] OR "Levofloxacin" OR "Fluoroquinolones" [mesh] OR "Fluoroquinolones" OR "Amoxicillin" [mesh] OR "Amoxicillin" OR "Ciprofloxacin" [mesh] OR "Ciprofloxacin" OR "Cephalexin" [mesh] OR "Cephalexin" OR "Tetracycline" [mesh] OR "Tetracycline"
	#3 #1 AND #2
Embase	#1 ('sars-related coronavirus'/exp OR 'sars-related coronavirus' OR 'covid 19' OR 'covid-19' OR 'novel coronavirus' OR 'sars-cov2' OR 'sars-ncov' OR 'sars-cov-2') AND [embase]/lim
	#2 'tetracycline'/exp OR 'cefalexin'/exp OR 'ciprofloxacin'/exp OR 'amoxicillin'/exp OR 'quinolone derivative'/exp OR 'levofloxacin'/exp OR 'cefepime'/exp OR 'ceftriaxone'/exp OR 'vancomycin'/exp OR 'antibiotic agent'/exp OR 'azithromycin'/exp OR 'Antibiotics' OR 'Antibiotic'
	#3 #1 AND #2
Cochrane	# 1 MeSH descriptor: [SARS Virus] explode all trees
	#2 MeSH descriptor: [SARS Virus] explode all trees
	#3 "Covid-19" OR (COVID) OR (Coronavirus) OR (SARS-CoV-2) OR
	(Coronaviruses) OR (Deltacoronavirus) OR (Deltacoronaviruses) OR
	"Munia coronavirus HKU13" OR (Coronavirus HKU15) OR
	(Coronavirus, Rabbit) OR (Rabbit Coronavirus) OR (Coronaviruses,
	Rabbit) OR (Rabbit Coronaviruses) OR "Bulbul coronavirus HKU11"
	OR "Thrush coronavirus HKU12"
	#4 #1 OR #2 OR #3
	#5 MeSH descriptor: [Anti-Bacterial Agents] explode all trees
	#6 MeSH descriptor: [Azithromycin] explode all trees
	#7 MeSH descriptor: [Azithromycin] explode all trees
	#8 MeSH descriptor: [Ceftriaxone] explode all trees
	#9 MeSH descriptor: [Cefepime] explode all trees
	#10 MeSH descriptor: [Cefepime] explode all trees
	#11 MeSH descriptor: [Fluoroquinolones] explode all trees
	#12 MeSH descriptor: [Amoxicillin] explode all trees
	#13 MeSH descriptor: [Ciprofloxacin] explode all trees
	#14 MeSH descriptor: [Cephalexin] explode all trees
	#15 MeSH descriptor: [Tetracyclines] explode all trees
	#16 MeSH descriptor: [Doxycycline] explode all trees
	#17 #5 OR #6 OR #7 OR #8 OR #9 OR #10 OR #11 OR #12 OR #13 OR #14 OR #15 OR #16
	#18 #4 AND #17
meDrxiv	(SARS CoV OR SARS CoV 2 OR Covid-19) AND ("Anti-Bacterial Agents" OR antibiotics)
OpenGrey	(covid19 OR SARS-CoV2 OR SARS-CoV OR MERS-CoV OR severe acute respiratory syndrome coronavirus OR middle east respiratory syndrome coronavirus) AND antibiotics
ClinicalTrials.gov	COVID 19 OR Covid-19 OR SARS-CoV 2 OR SARS-CoV-2 OR nCoV 2019 OR severe acute respiratory syndrome coronavirus 2 /

## Apêndice 3 - Avaliação da certeza da evidência, de acordo com o sistema Grading of Recommendations Assessment, Development and Evaluation (GRADE)

**Pergunta 1 -** Cloroquina e/ou hidroxicloroquina comparadas a tratamento convencional em paciente com infecção por COVID-19

**Bibliografia**: Chen et al., 2020^(1)^; Chen et al., 2020^(2)^; Tang et al., 2020^(3)^; Gautret et al., 2020^(4)^; Mercuro et al., 2020^(5)^; Geleris et al., 2020^(6)^

**Table:**

Avaliação da certeza							Número de pacientes		Efeito		Certeza	Importância
Número dos estudos	Delineamento do estudo	Risco de viés	Inconsistência	Evidência indireta	Imprecisão	Outras considerações	Cloroquina e/ou hidroxicloroquina	Tratamento convencional	Relativo (IC95%)	Absoluto (IC95%)		
Mortalidade relacionada com COVID-19 (seguimento: média de 14 dias)												
1	Ensaios clínicos randomizados	Muito grave*	Não grave	Não grave	Grave^†^	Nenhum	Os autores não relataram mortes nos dois grupos aos 14 dias de acompanhamento				⨁◯◯◯ Muito baixa	Crítico
**Tempo até a intubação ou morte (seguimento: mediana de 22,5 dias)**												
1	Estudo observacional	Grave^‡^	Não grave	Não grave	Grave^§^		262/811 (32,3%)	84/565 (14,9%)	HR = 1,00 (0,76 para 1,32)	0 menos por 1 (de 66 menos para 79 mais)	⨁⨁◯◯ Baixa	Crítico
**Eventos adversos - quaisquer (seguimento: média de 14 dias)**												
3	Ensaios clínicos randomizados	Muito grave^¶^	Não grave	Não grave	Grave^||^	Nenhum	Chen et al.,2020^(1)^: houve três eventos adversos menores no grupo controle (aumento da creatinina, anemia e elevação do aspartato aminotransferase) e quatro eventos adversos menores no grupo hidroxicloroquina (diarreia: 2; interrupção do tratamento devido à deterioração do estado clínico: 1; e elevação do aspartato aminotransferase: 1) Chen et al., 2020^(2)^: dois eventos adversos menores no grupo hidroxicloroquina: rash cutâneo (1) e cefaleia (1) Tang et al., 2020^(3)^: 30% dos participantes no grupo hidroxicloroquina apresentaram eventos adversos e 8,8% no grupo controle. Evento adverso mais comum foi diarreia (10%)				⨁◯◯◯ Muito baixa	Crítico
**Desfecho laboratorial: negativação de detecção viral (PCR)**												
2	Ensaios clínicos randomizados	Muito grave^¶^	Não grave	Não grave^#^	Grave^††^	Nenhum	51/90 (56,7%)	54/90 (60,0%)	RR 0.94 (0,78 para 1,13)	36 menos por 1.000 (de 132 menos para 78 mais)	⨁⨁◯◯ Muito baixa	Importante
**Desfecho laboratorial: tempo para negativação de detecção viral (PCR) (seguimento: média de 7 dias)**												
2	Ensaios clínicos randomizados	Muito grave^¶^	Não grave	Não grave	Grave^††^	Nenhum	Chen et al., 2020^(2)^: não foram relatados dados suficientes para análises. Duração média de infecção no grupo hidroxicloroquina foi de 4 dias (primeiro quartil = 1; terceiro quartil = 9) *versus* 2 dias (primeiro quartil = 1; terceiro quartil = 4) no grupo controle Tang et al., 2020^(3)^: hidroxicloroquina: mediana 8 dias. Controle: mediana 7 dias (HR = 0,846; IC95% 0,58 - 1,23; p = 0,341).				⨁⨁◯◯ Muito baixa	Importante
**Eventos adversos: prolongamento QT**												
1	Estudo observacional	Grave^‡‡^	Não grave	Não grave	Grave^§§^	Nenhum	7/37 pacientes (19%) dos pacientes que receberam hidroxicloroquina (monoterapia) desenvolveram intervalo QT ≥ 500 milissegundos. Três pacientes (3%) tiveram mudança em QTc ≥ 60 milissegundos (Mercuro et al, 2020^(5)^)				⨁⨁◯◯ Muito baixa	Crítico

IC95% - intervalo de confiança de 95%; PCR - reação da cadeia da polimerase; HR - *hazard ratio*.

* Desfecho duro, não influenciado pelo delineamento aberto do estudo. Para eventos adversos, devemos considerar o alto risco de viés de desempenho. Risco pouco claro de viés de detecção e seleção;

† Estudo pequeno, nenhum evento foi observado nos dois grupos;

‡ risco de viés grave relacionado à seleção dos participação e classificação das intervenções;

§ intervalo de confiança amplo;

¶ alto risco de viés de desempenho. Risco pouco claro de viés de detecção e seleção;

|| pequeno tamanho amostral (92 pacientes), poucos eventos;

# o ensaio clínico não randomizado também incluído nesta revisão [Gautret 2020^(4)^] relatou taxa de negativação de carga viral de 70% do grupo hidroxicloroquina *versus* 12,5% no grupo controle (p = 0,001, após 6 dias);

** pequeno tamanho amostral, intervalo de confiança amplo, incluindo benefício e malefício importante;

†† pequeno tamanho amostral, poucos eventos;

‡‡ estudo não randomizado, não há comparador, mas assume-se que a taxa de prolongamento QT na ausência de tratamento tenha sido baixa;

§§ estudo com n pequeno.

**Pergunta 2 -** Hidroxicloroquina/cloroquina associada à azitromicina comparada a não utilizar em paciente com infecção por COVID-19

**Bibliografia:** Borba et al., 2020^(7)^; Million et al., 2020^(8)^; Lane et al., 2020^(9)^; Gautret et al., 2020^(10)^; Molina et al., 2020^(11)^; Chorin et al., 2020^(12)^; Columbia University Kidney Transplant Program, 2020^(13)^; Gabriels et al., 2020^(14)^; Ramireddy et al., 2020^(15)^; Chang et al., 2020^(16)^; Gautret et al., 2020^(4)^

**Table:**

Avaliação da certeza							Número de pacientes		Efeito		Certeza	Importância
Número dos estudos	Delineamento do estudo	Risco de viés	Inconsistência	Evidência indireta	Imprecisão	Outras considerações	Hidroxicloroquina/cloroquina + azitromicina	Não utilizar	Relativo (IC95%)	Absoluto (IC95%)		
**Negativação de detecção viral (PCR)**												
3	Estudo observacional	Grave*	Não grave	Não grave	Não grave	Nenhum	Million et al., 2020^(8)^: dos 1.061 pacientes avaliados, após 10 dias de tratamento com hidroxicloroquina + azitromicina, a persistência do derramamento viral foi observada em 47 pacientes (4,4%) Gautret et al.; 2020^(4)^: o grupo hidroxicloroquina + azitromicina teve 100% de pacientes negativados (n = 6/6) em comparação ao grupo hidroxicloroquina 57,1% (n = 8/14) Gautret et al., 2020^(10)^: dos 80 pacientes, após o tratamento hidroxicloroquina + azitromicina, observou-se queda rápida da carga viral nasofaríngea, com 83% de negativos no sétimo dia e 93% no oitavo dia^†^				⨁◯◯◯ Muito baixa	Importante
**Mortalidade cardiovascular**												
1	Estudo observacional	Não grave	Não grave	Grave^‡^	Não grave	Forte associação	Lane et al., 2020^(9)^: análise restrospectiva com 130 mil pacientes com artrite reumatoide mostrou risco de morte cardiovascular 119% maior em 30 dias (HR = 2,19; 1,22 - 3,94) com a hidroxicloroquina + azitromicina em comparação com hidroxicloroquina + azitromicina + amoxicilina				⨁⨁◯◯ Baixa	Crítico
**Mortalidade por todas as causas**												
6	Estudo observacional	Grave^§^	Não grave	Não grave	Não grave	Nenhum	Efeito combinado de seis estudos observacionais (Million et al., 2020^(8)^, Gautret et al., 2020^(10)^, Molina et al., 2020^(11)^, Chorin et al., 2020^(12)^, Columbia University Kidney Transplant Program, 2020^(13)^, Borba et al., 2020^(7)^) mostrou 35 mortes em um total de 1.342 pacientes incluídos				⨁◯◯◯ Muito baixa	Crítico
**Morbidade cardiovascular (arritmias, fibrilação atrial, alongamento QTc e insuficiência cardíaca)**												
6	Estudo observacional	Não grave	Não grave	Grave*	Não grave	Forte associação	Lane et al., 2020^(9)^: análise restrospectiva com 130 mil pacientes com artrite reumatoide mostrou aumento no risco de angina (HR = 1,15; IC95% 1,05- 1,26) e insuficiência cardíaca (HR = 1,22; IC95% 1,02-1,45) com associação de hidroxicloroquina + azitromicina Million et al., 2020^(8)^: dos 1.061 pacientes utilizando hidroxicloroquina + azitromicina, nenhum apresentou toxicidade cardíaca Borba et al., 2020^(7)^: 11/73 pacientes tratados com alta e baixa doses de cloroquina + ceftriaxona + azitromicina apresentaram intervalo QTc > 500ms Chorin et al., 2020^(12)^: dos 84 pacientes tratados utilizando hidroxicloroquina + azitromicina, 30% dos pacientes apresentaram aumento de QTc em mais de 40ms. Em 11% dos pacientes, o QTc aumentou para > 500ms Chang 2020^(16)^: ao longo de 295 pacientes-dia, houve 28 alertas urgentes para 18 (15,4%) pacientes. A fibrilação atrial com resposta ventricular rápida foi a mais comum (15, 53,6%). Houve cinco (17,9%) alertas para QTc> 500ms Ramireddy et al., 2020^(15)^: dos 98 pacientes incluídos no estudo, 61 pacientes fizeram uso de hidroxicloroquina + azitromicina. As alterações no QTc foram mais altas com a combinação dos dois fármacos, porém não significativas, quando comparadas com o grupo de pacientes que receberam só azitromicina (n=27)^¶^				⨁⨁◯◯	Crítico

IC95% - intervalo de confiança de 95%; PCR - reação da cadeia da polimerase; HR - *hazard ratio*.

* Gautret et al., 2020^(4)^ possui risco sério de viés para vários domínios considerados na avaliação;

† o estudo Lane et al., 2020^(9)^ incluiu pacientes com artrite reumatoide;

‡ série de casos com dez pacientes. Após o tratamento com a combinação de hidroxicloroquina (600mg) + azitromicina (500 mg no primeiro dia, 250 nos próximos 4 dias), oito pacientes (80%; intervalo de confiança de 95% 49-94) apresentaram detecção viral positiva após avaliação com reação em cadeia da polimerase (Molina et al., 2020^(11)^);

§ Borba, et al, 2020^(7)^ avaliaram hidroxicloroquina 600mg 2 vezes ao dia *versus* hidroxicloroquina 450mg 2 vezes ao dia (ambos os braços com associação com azitromicina), havendo interrupção precoce no grupo de dose alta por aumento de mortalidade;

¶ Gabriels et al., 2020: mulher de 72 anos com história de fibrilação atrial recebendo hidroxicloroquina + azitromicina realizou monitoramento de eletrocardiograma duas vezes por dia por telemetria. No segundo dia, depois da primeira dose do medicamento, paciente apresentou um episódio de arritmia. Paciente foi medicada e retornou ao ritmo sinusal. Seu tratamento com hidroxicloroquina + azitromicina foi continuado, sem mais eventos arrítmicos apresentando uma QTc estável; Molina et al., 2020^(11)^: em 1/11 paciente, a combinação de hidroxicloroquina (600mg) + azitromicina (500 mg no primeiro dia, 250 nos próximos 4 dias) foi descontinuada após 4 dias devido a um prolongamento do intervalo QT de 405ms antes do tratamento para 460 e 470ms após a combinação

**Pergunta 3 -** Oseltamivir comparado a não utilizar em paciente com infecção por COVID-19

**Bibliografia**: Liu et al., 2020^(17)^

**Table:**

Avaliação da certeza							Número de pacientes		Efeito		Certeza	Importância
Número dos estudos	Delineamento do estudo	Risco de viés	Inconsistência	Evidência indireta	Imprecisão	Outras considerações	Oseltamivir	Não utilizar	Relativo (IC95%)	Absoluto (IC95%)		
**Melhora radiológica**												
1	Estudo observacional	Grave*	Não grave	Não grave	Não grave	Nenhum	Oseltamivir (55 pacientes) *versus* não uso (271 pacientes): 41,18% *versus* 43,34%				⨁◯◯◯ Muito baixa	Importante
**Mortalidade**												
1	Estudo observacional	Grave*	Não grave	Não grave	Não grave	Nenhum	8/66 (12,1%)	84/516 (16,3%)	RC = 0,713 (0,282 para 1,589)	41 menos por 1.000 (de 111 menos para 73 mais)	⨁◯◯◯ Muito baixa	Crítico

IC95% - intervalo de confiança de 95%; RC - razão de chance.

* Alto risco de viés devido a: seleção, comparabilidade, verificação da exposição, controle de confundidores e aferição dos desfechos.

**Pergunta 4 -** Lopinavir associado a ritonavir comparado a cuidados padrão em paciente com infecção por COVID-19

**Bibliografia**: Cao et al., 2020^(18)^; Li et al., 2020^(19)^; Deng et al., 2020^(20)^; Ye et al., 2020^(21)^; Zhu et al., 2020^(22)^; Shi et al., 2020^(23)^; Liu et al., 2020^(17)^; Sun et al., 2020^(24)^

**Table:**

Avaliação da certeza							Número de pacientes		Efeito		Certeza	Importância
Número dos estudos	Delineamento do estudo	Risco de viés	Inconsistência	Evidência indireta	Imprecisão	Outras considerações	Lopinavir/ritonavir	Cuidados padrão	Relativo (IC95%)	Absoluto (IC95%))		
**Melhora clínica**												
2	Ensaios clínicos randomizados	Grave*	Não grave	Não grave	Grave^†^	Nenhum	Cao et al.^(18)^, 2020: % de melhora clínica aos 14 dias, mediana - Lopinavir/ritonavir *versus* padrão: 45(45,5%) *versus* 30 (30,0%); diferença, 15,5%; (IC95% 2,2 - 28,8) Li et al., 2020^(19)^: Taxa de alívio de tosse - aos 14 dias: lopinavir/ritonavir: 17/19 (89,5%); controle: 5/6 (83,3%) Taxa de antipirese - aos 14 dias: lopinavir/ritonavir: 10/11 (90,9%); controle: 2/2 (100%) Taxa de deterioração para estado grave - lopinavir/ritonavir: 8/21 (38,1%); controle: 1/7(14,3%) Melhora na tomografia computadorizada de tórax - aos 14 dias: lopinavir/ritonavir: 16/19 (84,2%); controle: 6/6 (100%)^‡,§,¶,||^				⨁⨁◯◯ Baixa	Importante
**Mortalidade em 28 dias (seguimento: 28 dias)**												
1^#^	Ensaios clínicos randomizados	Grave*	Não grave	Não grave	Grave^†^	Nenhum	19/99 (19,2%)	25/100 (25,0%)	HR = 0,77 (0,45 para 1,30)	51 menos por 1.000 (de 129 menos para 62 mais)	⨁⨁◯◯ Baixa	Crítico
**Tempo de hospitalização em UTI (seguimento: 28 dias)**												
1	Ensaios clínicos randomizados	Grave*	Não grave	Não grave	Grave**	Nenhum	Estadia em UTI (mediana, dias) lopinavir/ritonavir *versus* padrão: 6 *versus* 11; diferença, -5 dias (IC95% -9 - 0)				⨁⨁◯◯ Baixa	Importante
**Tempo de hospitalização (seguimento: 28 dias)**												
1	Ensaios clínicos randomizados	Grave*	Não grave	Não grave	Grave^††^	Nenhum	Lopinavir *versus* padrão: 12 *versus* 14; diferença, 1 dia; (IC95% 0 - 3)^‡‡^				⨁⨁◯◯ Baixa	Importante
**Carga viral**												
2	Ensaios clínicos randomizados	Grave*	Não grave	Não grave	Grave^†^	Nenhum	Cao et al., 2020^(18)^: Carga viral em 14 dias (log10 cópias/mL - DP) - lopinavir/ritonavir *versus* padrão: 4,4 ± 2,0 *versus* 3,7 ± 2,1 % de pacientes com RNA SARS-CoV-2 - lopinavir/ritonavir *versus* padrão: dia 5, 34.5% *versus* 32.9%; dia 10, 50.0% *versus* 48.6%; dia 14, 55.2% *versus* 57.1%; dia 21, 58.6% *versus* 58.6%; e dia 28, 60.3% *versus* 58.6% Li et al., 2020^(19)^: Tempo de negativação RNA SARS-CoV-2 - lopinavir/ritonavir *versus* controle: 8,7 ± 6,0 dias *versus* 7,0 ± 5,9 dias; Taxas de negativação após 7 dias: lopinavir/ritonavir *versus* controle: 42,9% (21/9) *versus* 71,4% (5/7); Taxas de negativação após 14 dias: lopinavir/ritonavir *versus* controle: 76,2% (16/21) *versus* 71,4% (5/7)^§§¶¶^				⨁⨁◯◯ Baixa	Importante
**Eventos adversos**												
2	Ensaios clínicos randomizados	Grave*	Grave^||||^	Não grave	Grave^##^	Nenhum	Cao et al.^(18)^: Evento adverso grau III ou IV, lopinavir/ritonavir *versus* padrão, n (%) qualquer: 20 (21,1) *versus* 11 (11,1); linfopenia: 12 (12,6) *versus* 5 (5,1) Evento grave: 17 (17,9) *versus* 31 (31,3); falha respiratória ou SARA: 12 (12,6) *versus* 27 (27,3); dano renal agudo: 2 (2,1) *versus* 5 (5,1); infecção secundária: 1(1,1) *versus* 6 (6,1). Li et al., 2020^(19)^: Grupo lopinavir/ritonavir: 5 (23,8%) apresentaram eventos adversos. Eventos: diarreia (3/21;14,3%), perda de apetite (2/21; 9,5%) e elevação da alanina aminotransferase > de 2,5 (1/21; 4,8%). Nenhum evento adverso ocorreu no grupo arbidol ou no grupo controle***				⨁⨁◯◯ Muito baixa	Crítico

IC95% - intervalo de confiança de 95%; HR - *hazard ratio*; UTI - unidade de terapia intensiva; DP - desvio padrão; SARS-CoV-2 - síndrome respiratória aguda grave; SARA - síndrome de angústia respiratória aguda.

* É possível que o conhecimento da atribuição do tratamento possa ter influenciado na tomada de decisão clínica, o que poderia afetar as medidas da escala ordinal utilizadas. Ademais, inicialmente havia maior carga viral nos pacientes do grupo lopinavir/ritonavir, o que pode ter dificultado os resultados de redução da carga viral;

† para desfechos com potencial risco e benefício (não significantes), o GRADE recomenda que valores abaixo do limiar de 25% para benefício ou risco (ou seja 1,25 ou 0,75, para medidas dicotômicas) devam ser classificados como imprecisos;

‡ Cao et al., 2020^(18)^: tempo até a melhora clínica (melhora de 2 pontos na escala) - *hazard ratio* = 1,31; intervalo de confiança de 95% 0,95 - 1,85; p = 0,09; Tempo até a deterioração (piora de 2 pontos na escala) - *hazard ratio* = 1,01; intervalo de confiança de 95% 0,76 - 1,34;

§ dois estudos observacionais: Deng et al., 2020^(20)^ - estudo comparando lopinavir/ritonavir *versus* umifenovir + lopinavir/ritonavir apresenta dados para melhora da pneumonia por tomografia computadorizada de tórax: lopinavir/ritonavir *versus* umifenovir + lopinavir/ritonavir: 5 (29%) *versus* 11 (69%). Ye et al., 2020^(21)^ - estudo comparando lopinavir/ritonavir + associações (interferon e umifenovir) *versus* associações (interferon e umifenovir) apresenta dados de tempo de melhora na temperatura corporal: grupo teste (n=42) = 4,8 ± 1,94 dias *versus* grupo controle (n=5) = 7,3 ± 1,53 dias, p = 0,0364;

¶ Shi et al. 2020^(23)^: coorte retrospectiva com 184 pacientes divididos em sete grupos de antivirais. Não foi demonstrada diferença significativa na alteração do volume de pneumonia nos resultados de imagem por tomografia computadorizada entre os grupos (p = 0,151). Não houve diferença significativa na proporção de resolução de pneumonia entre os grupos após o ciclo de tratamento de 5 dias (p = 0,116);

|| Liu et al., 2020. Coorte retrospectiva com 504 pacientes; 257 pacientes receberam umifenovir; 66 pacientes receberam oseltamivir;259 pacientes receberam lopinavir/ritonavir. Em relação à redução média do tamanho da lesão por tomografia computadorizada (n=326 sobreviventes): umifenovir (209 pacientes) *versus* não uso (117 pacientes): 46,43% (desvio padrão ± 29,00) *versus*  36,80% (desvio padrão ± 24,95); oseltamivir (55 pacientes) *versus* não uso (271 pacientes): 41,18% *versus* 43,34%; lopinavir/ritonavir (186 pacientes ) *versus* não uso (140 pacientes): 37,26% *versus* 50,56%;

# Liu et al., 2020^(17)^: coorte retrospectiva com 504 pacientes de 3 hospitais com COVID-19; 257 pacientes receberam umifenovir; 66 pacientes receberam oseltamivir; 259 pacientes receberam lopinavir/ritonavir. Em relação à mortalidade: umifenovir x não uso: 7,0% *versus* 24,7% (razão de chance: 0,230; intervalo de confiança de 95% 0,124 - 0,411); oseltamivir *versus* não uso: 12,2% *versus* 16,21%; razão de chance: 0,713; intervalo de confiança de 95% 0,282 - 1,589); lopinavir/ritonavir *versus* não uso: 14,29% *versus* 17,14%; razão de chance: 0,806; intervalo de confiança de 95% 0,483- 1,341);

** desfecho envolve intervalo de confiança amplo, variando de não efeito à não diferença estatística;

†† o intervalo de confiança não é largo, mas existe certa imprecisão clínica, pois o dado é não significativo, ou seja, pode não ter diferença na estadia entre grupo ou ter dias a mais. Dias a mais de unidade de terapia intensiva geram um impacto enorme, clínica e financeiramente;

‡‡ Shi et al., 2020^(23)^: coorte retrospectiva de 184 pacientes divididos em sete grupos de antivirias. Não houve diferença significativa no tempo de internação entre os grupos (p = 0,355);

§§ dois estudos observacionais: Deng et al., 2020^(20)^ - estudo comparando lopinavir/ritonavir *versus* Umifenovir + lopinavir/ritonavir apresenta dados para: carga viral zerada em 7 dias: lopinavir/ritonavir *versus* umifenovir + lopinavir/ritonavir: 6 (35%) *versus* 12 (75%); p<0,05; e carga viral zerada em 14 dias: lopinavir/ritonavir *versus* umifenovir + lopinavir/ritonavir: 9 (53%) *versus* 15 (94%); p < 0,05; Ye et al., 2020^(21)^ - estudo observacional comparando lopinavir/ritonavir + associações (interferon e umifenovir) *versus* associações (interferon e umifenovir) apresenta dados para: tempo médio de negativação RNA síndrome respiratória aguda grave: lopinavir/ritonavir *versus* controle: 7,8 ± 3,09 dias *versus* 12,0 ± 0,82 dias; p = 0,0219;

¶¶ Zhu et al., 2020^(22)^: coorte restrspectiva que inclui 55 pacientes com diagnóstico de COVID-19; 34 pacientes receberam lopinavir/ritonavir. No sétimo dia após admissão, a carga viral foi indetectável em 50% dos pacientes que receberam umifenovir e em 23,5% dos pacientes tratados com lopinavir/ritonavir. No 14º dia após a admissão, a carga viral foi indetectável em todos os pacientes do grupo umifenovir e detectável em 44,1% dos pacientes que receberam lopinavir/ritonavir;

|||| Um dos estudos não demonstra eventos adversos no grupo controle;

## são mostrados eventos adversos que ocorreram em mais de um paciente após a randomização até o dia 28. Alguns pacientes tiveram mais de um evento adverso. Não houve nenhuma intenção do estudo em diferenciar os grupos estatisticamente, apesar de, para alguns eventos adversos, as diferenças entre os grupos serem de quase 50%;

*** Sun et al., 2020^(24)^: coorte retrospectiva com 217 pacientes internados com COVID-19. Um total de 94 reações adversas ao medicamento foi identificada em 82 pacientes. A taxa de incidência foi de 37,8%: 119 (54,8%) pacientes usaram umifenovir; 179 (82,5%) pacientes usaram lopinavir/ritonavir; 37 (17,1%) pacientes usaram cloroquina.

**Pergunta 5 -** Corticoides comparados a não utilizar em paciente com infecção por COVID-19

**Contexto**: Devemos utilizar corticoides no paciente com infecção por COVID-19?

**Bibliografia**: Zhou et al., 2020^(25)^; Wu et al., 2020^(26)^; Guan et al., 2020^(27)^; Shang et al., 2020^(28)^; Cao et al.,2020^(29)^; Li et al., 2020^(30)^; Xu et al.^(31)^, 2020; Zha et al., 2020^(32)^; Lu et al., 2020^(33)^; Wang et al., 2020^(34)^

**Table:**

Avaliação da certeza							Impacto	Certeza	Importância
Número dos estudos	Delineamento do estudo	Risco de viés	Inconsistência	Evidência indireta	Imprecisão	Outras considerações			
**Melhora de quadro respiratório**									
2	Estudo observacional	Grave*^†^	Grave^‡^	Não grave	Não grave^§^	Nenhum	Zhou et al., 2020^(25)^: após introdução de corticosteroides, pacientes com hipoxemia e SDRA por COVID-19 apresentaram - Aumento significativo de SpO_2_ nos dias 3 e 9 (p=0,030 e p=0,012, respectivamente) - Aumento nos valores de PaO_2_/FiO_2_ no dia 9 (p=0,034) Guan et al., 2020^(27)^: em série de casos com 1.099 pacientes com COVID-19, 8,3% dos pacientes necessitaram de ventilação mecânica após o início da terapia com corticosteroides	⨁◯◯◯ Muito baixa	Importante
**Alteração em exames laboratoriais**									
2	Estudo observacional	Grave*^†^	Grave^‡^	Não grave	Não grave^§^	Nenhum	Zhou et al., 2020^(25)^: após introdução de corticosteroides, pacientes com hipoxemia e SDRA por COVID-19 apresentaram: - Redução dos valores de PCR nos dias 4 e 10 (p = 0,003 e p = 0,035, respectivamente) - Redução nos valores de fibrinogênio no dia 4 (p = 0,014) - Redução nos valores de dímero D nos dias 4, 7 e 10 (p = 0,019, p = 0,027 e p = 0,047, respectivamente) Shang et al., 2020^(28)^: pacientes que sobreviveram à COVID-19 grave que utilizaram corticosteroides tiveram aumento da contagem de glóbulos brancos, neutrófilos e linfócitos ao final do tratamento. Pacientes que não utilizaram corticosteroides apresentaram aumento na contagem de glóbulos brancos e linfócitos. Os grupos que utilizaram e não utilizaram corticosteroides foram diferentes apenas na contagem de linfócitos pré-tratamento. Pacientes com COVID-19 que evoluíram a óbito que utilizaram corticosteroides tiveram aumento da contagem de glóbulos brancos, neutrófilos e linfócitos e na proporção de neutrófilos e linfócitos ao final do tratamento. Não houve diferença com significância estatística no pré e pós-tratamento no grupo que não recebeu corticosteroides e entre os grupos que receberam e não receberam o medicamento Xu et al.,2020^(31)^: estudo avaliou 113 (28% sintomas severos) pacientes, dos quais 56,6% utilizaram corticoides. O tratamento com corticosteroides foi relacionado a tempo prolongado para *clearance* viral (15/37 pacientes com *clerance* precoce, 40,5% *versus* 49/76 com *clerance* tardio, 64,5%; p = 0,025; RC = 1,38; IC95% 0,52 - 3,65; p = 0,519) Zha et al.,2020^(32)^: 11/31 pacientes com sintomas moderados receberam prednisolona (40mg 1 a 2/dia, por média de 5 dias). Não houve diferenças estatisticamente significativas nos resultados sobre carga viral ou clínicos (duração de sintomas, tempo hospitalização, comprometimento renal ou hepático) entre os pacientes que receberam e aqueles que não receberam corticosteroide	⨁◯◯◯ Muito baixa	Importante
**Mortalidade**									
3	Estudo observacional	Grave*^†^	Grave^‡^	Não grave	Não grave^§^	Nenhum	Guan et al., 2020^(27)^: em coorte retrospectiva com 201 pacientes com COVID-19, 62 (30,8%) receberam corticosteroides. Neste estudo, o uso de metilprednisolona aparentemente reduziu o risco de morte em pacientes com SDRA (HR = 0,38; IC95% 0,20 - 0,72; p = 0,003) (Wu et al., 2020(26)). Em estudo com 1.099 pacientes internados por COVID-19, 18,6% utilizaram glicocorticoides. Destes, 2,5% evoluíram a óbito Cao et al.,2020^(29)^: em coorte retrospectiva com 416 pacientes internados por COVID-19, 51 faleceram. Destes, 84% receberam corticosteroides (metilprednisolona, acetato de prednisona e dexametasona) (Shang et al., 2020^(29)^). Série de casos avaliou 102 pacientes, na qual 50% utilizaram metilpredisolona. Não houve diferença entre os grupos que sobreviveram e não sobreviveram em relação ao uso de terapia com glicocorticoides (p = 0,184) Li et al., 2020^(30)^: coorte retrospectiva, na qual 341/548 (62,2%) pacientes utilizaram corticosteroides sistêmicos durante internação, com duração média de 4 dia, média equivalente a 200 mg de prednisona) - A administração de altas doses de corticosteroides (≥ 1 mg/kg/dia prednisona) foi fator de risco para mortalidade (HR = 3,5; IC95% 1,8 - 6,9) durante a hospitalização Lu et al., 2020^(33)^: 151/244 (62%) receberam tratamento adjuvante com corticosteroides (equivalente à hidrocortisona: 100-800mg/d) por, em média, 8 dias. As disfunções de múltiplos órgãos foram mais comuns no grupo em uso de esteroide do que no grupo não esteroide - Uso de corticosteroides não mostrou associação com aumento de chance para mortalidade geral (RC = 1,05; IC95% -1,92-2,01) - Comparando pacientes em uso de corticoide *versus* os que não utilizaram, cada aumento de 10mg de hidrocortisona foi associado a um risco adicional de 4% de mortalidade (HR ajustado = 1,04; IC95% 1,01-1,07) Wang et al., 2020^(34)^: em coorte retrospectiva, 73/115 (63,5%) receberam tratamento com corticosteroides, sendo 31 (51,7%) pacientes não críticos e 42 (76,4%) pacientes críticos. O grupo corticosteroide teve maior número de admissões na UTI ou mortalidade (24/73; 32,9% *versus* 5/42; 11,9%; p = 0,013) - RC de mortalidade ou admissão na UTI 2,155 (IC95% 0,493 - 9,427; p = 0,308)	⨁◯◯◯ Muito baixa	Crítico
Admissão em UTI									
1	Estudo observacional	Muito grave*	Não grave	Não grave	Não grave	Nenhum	Guan et al., 2020^(27)^: em 1.099 pacientes internados por COVID-19, 18,6% utilizaram glicocorticoides. Destes, 16,2% foram internados em UTI Wang et al., 2020^(34)^: em coorte retrospectiva, 73/115 (63,5%) receberam tratamento com corticosteroides, sendo 31 (51,7%) pacientes não críticos e 42 (76,4%) pacientes críticos. O grupo corticosteroide teve maior número de admissões na UTI ou mortalidade (24/73, 32,9% *versus* 5/42, 11,9%, p = 0,013) - RC de mortalidade ou admissão na UTI 2,155 (IC95% 0,493 - 9,427, p = 0,308).	⨁◯◯◯ Muito baixa	Crítico
Tempo de internação hospitalar									
1	Estudo observacional	Grave^†^	Não grave	Não grave	Não grave	Nenhum	Shang et al., 2020^(28)^: em coorte de 416 pacientes com COVID-19, observou-se que: -Sobreviventes comuns (corticosteroides *versus* não corticosteroides): dias de hospitalização indicados por mediana (intervalo interquartil): 12,0 (9,0 - 16,0) *versus* 10,0 (8,0 - 13,0), p < 0,05 -Sobreviventes críticos (corticosteroides *versus* não corticosteroides): dias de hospitalização indicados por mediana (intervalo interquartil): 14,0 (10,0 - 18,0) *versus* 11,0 (9,0 - 13,0), p < 0,05 -Óbitos (corticosteroides vs. não corticosteroides): dias de hospitalização indicados por mediana (intervalo interquartil): 11,0 (7,0 - 13,0) *versus* 11,5 (8,0 - 16,0), p > 0,05	⨁◯◯◯ Muito baixa	Crítico

SDRA - síndrome do desconforto respiratório agudo; SpO_2_ - Saturação de Oxigênio; PaO_2_/FiO_2_ - pressão parcial de oxigênio/fração inspirada de oxigênio; PCR - proteína C-reativa; RC - razão de chance; IC95% - intervalo de confiança de 95%; HR - *hazard ratio*; UTI - unidade de terapia intensiva.

* Alto risco de viés (série de casos);

† moderado risco de viés (NOS-Scale);

‡ alguns estudos mostram benefícios, outros riscos ou nulidade de efeito;

§ imprecisão gerada pela heterogeneidade dos dados.

**Pergunta 6 -** Tocilizumabe comparado a não utilizar em paciente com infecção por COVID-19

**Bibliografia**: Luo et al., 2020^(35)^; Xu et al., 2020^(36)^

**Table:**

Avaliação da certeza							Número de pacientes		Efeito		Certeza	Importância
Número dos estudos	Delineamento do estudo	Risco de viés	Inconsistência	Evidência indireta	Imprecisão	Outras considerações	Tocilizumabe	Não utilizar	Relativo (IC95%)	Absoluto (IC95%)		
**Mortalidade**												
2	Estudo observacional	Grave*	Não grave	Não grave	Grave^†^	Nenhum	Xu et al., 2020^(36)^: estudo relatou que, durante o tratamento com tocilizumabe, nenhuma morte foi relatada Luo et al., 2020^(35)^: estudo relatou 3 mortes entre 15 pacientes durante o tratamento com tocilizumabe				⨁◯◯◯ Muito baixa	Crítico
**Eventos adversos**												
1	Estudo observacional	Grave*	Não grave	Não grave	Grave^†^	Nenhum	Xu et al., 2020^(36)^: não relataram eventos adversos graves. No entanto, os pacientes que recebem tocilizumabe geralmente apresentam um risco aumentado de infecções graves (bacterianas, virais, infecções fúngicas invasivas e tuberculose) e reativação da hepatite B Foram relatados casos de anafilaxia, reações alérgicas graves, lesão hepática grave e insuficiência hepática e perfuração intestinal após a administração de tocilizumabe em pacientes sem COVID-19*				⨁◯◯◯ Muito baixa	Crítico
**Falha na melhora clínica (avaliado com: achados da tomografia computadorizada)**												
2	Estudo observacional	Grave*	Não grave	Não grave	Grave^†^	Nenhum	Xu et al., 2020^(36)^: dos 21 pacientes, 19 receberam alta hospitalar, sugerindo taxa de 9,5% de falha na melhora clínica nos achados da tomografia computadorizada Luo et al., 2020^(35)^: dos 15 pacientes, 3 morreram, 2 pacientes agravaram a doença e 9 estabilizaram clinicamente e 1 teve melhora clínica				⨁◯◯◯ Muito baixa	Importante

IC95% -intervalo de confiança de 95%. * Séries e relatos de casos; ^†^ poucos casos relatados. * Referências: 1. Genentech, Inc. ACTEMRA® (tocilizumab) injection, for intravenous or subcutaneous use. San Francisco, CA: Genentech, Inc; 2019.

**Pergunta 7 -** Heparinas comparadas a não utilizar em paciente com infecção por COVID-19

**Bibliografia**: Shi et al., 2020^(37)^; Tang et al., 2020^(38)^

**Table:**

Avaliação da certeza							Número de pacientes		Efeito		Certeza	Importância
Número dos estudos	Delineamento do estudo	Risco de viés	Inconsistência	Evidência indireta	Imprecisão	Outras considerações	Heparinas	Tratamento usual	Relativo (IC95%)	Absoluto (IC95%)		
**Tempo de internação hospital (avaliado com: dias)**												
1	Estudo observacional	Muito grave*	Não grave	Não grave	Muito grave^†^	Nenhum	Shi et al., 2020^(37)^ relataram tempo de internação hospitalar de 29 dias (17 - 42) no grupo heparina *versus* 27 dias (24 - 31) no grupo controle (p = 0,41)				⨁◯◯◯ Muito baixa	Importante
**Dímero D (avaliado com: variação a partir da linha de base, ng/dL)**												
1	Estudo observacional	Muito grave*	Não grave	Não grave	Muito grave^†^	Nenhum	Shi et al., 2020^(37)^ relataram variação dos níveis de dímero D de -2,85 ± 3,9 no grupo heparina *versus* -0,05 ± 0,85 no grupo controle (p = 0,002)				⨁◯◯◯ Muito baixa	Importante
**Interleucina 6 (avaliado com: variação a partir da linha de base, pg/mL)**												
1	Estudo observacional	Muito grave*	Não grave	Não grave	Muito grave^†^	Nenhum	Shi et al., 2020^(37)^ relataram variação dos níveis de interleucina 6 de -32,46 pg/mL ± 65, 97 no grupo heparina *versus* 14,96 pg/mL ± 151,09 no grupo controle (p = 0,031)				⨁◯◯◯ Muito baixa	Importante
**Linfócitos (avaliado com: variação a partir da linha de base, %)**												
1	Estudo observacional	Muito grave*	Não grave	Não grave	Muito grave^†^	Nenhum	Shi et al., 2020^(37)^ relataram variação na porcentagem a partir da linha de base de 11,1 ± 9,50 no grupo heparina *versus* 3,08 ± 9,66 no grupo controle (p = 0,011)				⨁◯◯◯ Muito baixa	Importante
**Mortalidade após 28 dias, todos os participantes**												
1	Estudo observacional	Muito grave*	Não grave	Não grave	Muito grave^‡^	Nenhum	Tang et al., 2020^(38)^ relataram mortalidade de 30,3% no grupo heparina *versus* 29,7% no grupo controle (p = 0,910)				⨁◯◯◯ Muito baixa	Crítico
**Mortalidade após 28 dias, participantes com dímero D > seis vezes o limite superior de normalidade**												
1	Estudo observacional	Muito grave*	Não grave	Não grave	Muito grave^‡^	Nenhum	Tang et al.^(38)^, relataram mortalidade de 32,8% no grupo heparina *versus* 52,4% no grupo controle (p = 0,017)				⨁◯◯◯ Muito baixa	Crítico
**Mortalidade após 28 dias, pacientes com escore SIC (coagulopatia induzida por sepse) ≥ 4**												
1	Estudo observacional	Muito grave*	Não grave	Não grave	Não grave^c^	Nenhum	Tang et al., 2020^(38)^ relataram mortalidade de 40% no grupo heparina *versus* 64,2% no grupo controle (p = 0,029)				⨁◯◯◯ Muito baixa	Crítico

IC95% - intervalo de confiança de 95%; SIC - *Sepsis-Induced Coagulopathy*.

* Risco crítico de viés avaliado pela ferramenta Robins-I. Estudo retrospectivo, sem controle de fatores de confusão;

† estudo único, com pequeno tamanho amostral;

‡ estudo único.

**Pergunta 8 -** Antibioticoterapia comparada a não utilizar em paciente com COVID-19 sem evidência de infecção bacteriana

Não foram encontrados estudos em paciente com COVID-19 sem evidência de infecção bacteriana

**REFERÊNCIAS**

1. Chen J, Liu D, Liu L, Liu P, Xu Q, Xia L, et al. [A pilot study of hydroxychloroquine in treatment of patients with moderate COVID-19. Zhejiang Da Xue Xue Bao Yi Xue Ban. 2020;49(2):215-9. Chinese.

2. Chen Z, Hu J, Zhang Z, Jiang S, Han S, Yan D, et al. Efficacy of hydroxychloroquine in patients with COVID-19: results of a randomized clinical trial. medRxiv. 2020:2020.03.22.20040758.

3. Tang W, Cao Z, Han M, Wang Z, Chen J, Sun W, et al. Hydroxychloroquine in patients with mainly mild to moderate coronavirus disease 2019: open-label, randomized, controlled trial. BMJ. 2020;369:m1849.

4. Gautret P, Lagier JC, Parola P, Hoang VT, Meddeb L, Mailhe M, et al. Hydroxychloroquine and azithromycin as a treatment of COVID-19: results of an open-label non-randomized clinical trial. Int J Antimicrob Agents. 2020 Mar 20:105949. Online ahead of print.

5. Mercuro NJ, Yen CF, Shim DJ, Maher TR, McCoy CM, Zimetbaum PJ, et al. Risk of QT interval prolongation associated with use of hydroxychloroquine with or without concomitant azithromycin among hospitalized patients testing positive for coronavirus disease 2019 (COVID-19). JAMA Cardiol. 2020 may 1. Online ahead of print.

6. Geleris J, Sun Y, Platt J, et al. Observational study of hydroxychloroquine in hospitalized patients with Covid-19. N Engl J Med. 2020 May 7. Online ahead of print.

7. Borba MG, Val FF, Sampaio VS, Alexandre MA, Melo GC, Brito M, Mourão MP, Brito-Sousa JD, Baía-da-Silva D, Guerra MV, Hajjar LA, Pinto RC, Balieiro AA, Pacheco AG, Santos JD Jr, Naveca FG, Xavier MS, Siqueira AM, Schwarzbold A, Croda J, Nogueira ML, Romero GA, Bassat Q, Fontes CJ, Albuquerque BC, Daniel-Ribeiro CT, Monteiro WM, Lacerda MV; CloroCovid-19 Team. Effect of high vs low doses of chloroquine diphosphate as adjunctive therapy for patients hospitalized with severe acute respiratory syndrome coronavirus 2 (SARS-CoV-2) infection: a randomized clinical trial. JAMA Netw Open. 2020;3(4):e208857.

8. Million M, Lagier JC, Gautret P, Fournier PE, Amrane S, Hocquart M, et al. Early treatment of COVID-19 patients with hydroxychloroquine and azithromycin: a retrospective analysis of 1061 cases in Marseille, France. Travel Med Infect Dis. 2020 May 5:101738. Online ahead of print.

9. Lane JC, Weaver J, Kostka K, Duarte-Salles T, Abrahao MT, Alghoul H, et al. Safety of hydroxychloroquine, alone and in combination with azithromycin, in light of rapid wide-spread use for COVID-19: a multinational, network cohort and self-controlled case series study. medRxiv. 2020:2020.04.08.20054551.

10. Gautret P, Lagier JC, Parola P, Hoang VT, Meddeb L, Sevestre J, et al. Clinical and microbiological effect of a combination of hydroxychloroquine and azithromycin in 80 COVID-19 patients with at least a six-day follow up: a pilot observational study. Travel Med Infect Dis. 2020;34:101663.

11. Molina JM, Delaugerre C, Le Goff J, Mela-Lima B, Ponscarme D, Goldwirt L, et al. No evidence of rapid antiviral clearance or clinical benefit with the combination of hydroxychloroquine and azithromycin in patients with severe COVID-19 infection. Med Mal Infect. 2020;50(4):384.

12. Chorin E, Dai M, Shulman E, Wadhwani L, Cohen RB, Barbhaiya C, et al. The QT interval in patients with SARS-CoV-2 infection treated with hydroxychloroquine/azithromycin. medRxiv. 2020:2020.04.02.20047050.

13. Columbia University Kidney Transplant Program. Early description of coronavirus 2019 disease in kidney transplant recipients in New York. J Am Soc Nephrol. 2020 Apr 21;ASN.2020030375. Online ahead of print.

14. Gabriels J, Saleh M, Chang D, Epstein LM. Inpatient use of mobile continuous telemetry for COVID-19 patients treated with hydroxychloroquine and azithromycin. HeartRhythm Case Rep. 2020 Apr 1. Online ahead of print.

15. Ramireddy A, Chugh HS, Reinier K, Ebinger J, Park E, Thompson M, et al. Experience with hydroxychloroquine and azithromycin in the COVID-19 pandemic: implications for QT interval monitoring. MedRxiv. 2020: 2020.04.22.20075671.

16. Chang D, Saleh M, Gabriels J, Ismail H, Goldner B, Willner J, et al. Inpatient use of ambulatory telemetry monitors for COVID-19 patients treated with hydroxychloroquine and/or azithromycin. J Am Coll Cardiol. 2020 Apr 18:S0735-1097(20)35009-9. Online ahead of print.

17. Liu Q, Fang X, Tian L, Chen X, Chung U, Wang K, et al. The effect of arbidol hydrochloride on reducing mortality of Covid-19 patients: a retrospective study of real world date from three hospitals in Wuhan. medRxiv. 2020: 2020.04.11.20056523.

18. Cao B, Wang Y, Wen D, Liu W, Wang J, Fan G, et al. A trial of lopinavir-ritonavir in adults hospitalized with severe Covid-19. N Engl J Med. 2020;382(19):1787-99.

19. Li Y, Xie Z, Lin W, Cai W, Wen C, Guan Y, et al. An exploratory randomized, controlled study on the efficacy and safety of lopinavir/ritonavir or arbidol treating adult patients hospitalized with mild/moderate COVID-19 (ELACOI). medRxiv. 2020:2020.03.19.20038984.

20. Deng L, Li C, Zeng Q, Liu X, Li X, Zhang H, et al. Arbidol combined with LPV/r versus LPV/r alone against corona virus disease 2019: a retrospective cohort study. J Infect. 2020 Mar 11;S0163-4453(20)30113-4. Online ahead of print.

21. Ye XT, Luo YL, Xia SC, Sun QF, Ding JG, Zhou Y, et al. Clinical efficacy of lopinavir/ritonavir in the treatment of Coronavirus disease 2019. Eur Rev Med Pharmacol Sci. 2020;24(6):3390-6.

22. Zhu Z, Lu Z, Xu T, Chen C, Yang G, Zha T, et al. Arbidol monotherapy is superior to lopinavir/ritonavir in treating COVID-19. J Infect. 2020 Apr 10;S0163-4453(20)30188-2. Online ahead of print.

23. Shi X, Lu Y, Li R, Tang Y, Shi N, Song F, et al. Evaluation of antiviral therapies for coronavirus disease 2019 (COVID-19) pneumonia in Shanghai, China. J Med Virol. 2020 Apr 16. Online ahead of print.

24. Sun J, Deng X, Chen X, Huang J, Huang S, Li Y, et al. Incidence of Adverse Drug Reactions in COVID-19 Patients in China: An Active Monitoring Study by Hospital Pharmacovigilance System. Clin Pharmacol Ther. 2020 Apr 23. Online ahead of print.

25. Zhou W, Liu Y, Tian D, Wang C, Wang S, Cheng J, et al. Potential benefits of precise corticosteroids therapy for severe 2019-nCoV pneumonia. Signal Transduct Target Ther. 2020;5(1):188.

26. Wu C, Chen X, Cai Y, Xia J, Zhou X, Xu S, et al. Risk factors associated with acute respiratory distress syndrome and death in patients with coronavirus disease 2019 pneumonia in Wuhan, China. JAMA Intern Med. 2020 Mar 13:e200994. Online ahead of print.

27. Guan WJ, Ni ZY, Hu Y, Liang WH, Ou CQ, et al. Clinical characteristics of coronavirus disease 2019 in China. N Engl J Med. 2020;382(18):1708-20.

28. Shang J, Du R, Lu Q, Wu J, Xu S, Ke Z, et al. The treatment and outcomes of patients with COVID-19 in Hubei, China: a multi-centered, retrospective, observational study. (2/26/2020). Available from: https://ssrn.com/abstract=3546060

29. Cao J, Tu WJ, Cheng W, et al. Clinical features and short-term outcomes of 102 patients with corona virus disease 2019 in Wuhan, China. Clin Infect Dis. 2020 Apr 2;ciaa243. Online ahead of print.

30. Li X, Xu S, Yu M, Wang K, Tao Y, Zhou Y, et al. Risk factors for severity and mortality in adult COVID-19 inpatients in Wuhan. J Allergy Clin Immunol. 2020 Apr 12:S0091-6749(20)30495-4. Online ahead of print.

31. Xu K, Chen Y, Yuan J, Yi P, Ding C, Wu W, et al. Factors associated with prolonged viral RNA shedding in patients with COVID-19. Clin Infect Dis. 2020 Apr 9:ciaa351. Online ahead of print.

32. Zha L, Li S, Pan L, Tefsen B, Li Y, French N, et al. Corticosteroid treatment of patients with coronavirus disease 2019 (COVID-19). Med J Aust. 2020;212(9):416-20.

33. Lu X, Chen T, Wang Y, Wang J, Yan F. et al. Adjuvant corticosteroid therapy for critically ill patients with COVID-19. Crit Care. 2020;24(1):241.

34. Wang D, Wang J, Jiang Q, Yang J, Li J, Gao C, et al. No clear benefit to the use of corticosteroid as treatment in adult patients with coronavirus disease 2019 : a retrospective cohort study. medRxiv. 2020:2020.04.21.20066258.

35. Luo P, Liu Y, Qiu L, Liu X, Liu D, Li J. Tocilizumab treatment in COVID-19: A single center experience. J Med Virol. 2020; Apr 6. Online ahead of print.

36. Xu X, Han M, Li T, Sun W, Wang D, Fu B, et al. Effective treatment of severe COVID-19 patients with tocilizumab. Proc Natl Acad Sci U S A. 2020;117(20):10970-5.

37. Shi C, Wang C, Wang H, Yang C, Cai F, Zeng F, et al. The potential of low molecular weight heparin to mitigate cytokine storm in severe COVID-19 patients: a retrospective clinical study. medRxiv. 2020:2020.03.28.20046144.

38. Tang N, Bai H, Chen X, Gong J, Li D, Sun Z. Anticoagulant treatment is associated with decreased mortality in severe coronavirus disease 2019 patients with coagulopathy. J Thromb Haemost. 2020;18(5):1094-9.

**Table:**

Intervenção	Principais reações adversas	Principais interações medicamentosas
Cloroquina e hidroxicloroquina	Prolongamento do intervalo QT, *torsades de pointes*, arritmias, retinopatia, cefaleia, tonturas, reações extrapiramidais, convulsões, comprometimento neuromuscular, alterações neuropsiquiátricas, trombocitopenia, pancitopenia, neutropenia, dor abdominal, náuseas, vômitos, diarreia, anorexia, hipoglicemia grave e insuficiência hepática aguda	Evitar associar a medicamentos que também prolonguem o intervalo QT (ondansetrona, domperidona, haloperidol, quinolonas, azitromicina, amiodarona etc).
Azitromicina	Náusea, vômito, diarreia, dor abdominal, prolongamento do intervalo QT, *torsades de pointes*, insuficiência hepática, reações dermatológicas e infusionais	Evitar associar a medicamentos que também prolonguem o intervalo QT (ondansetrona, domperidona, haloperidol, quinolonas, cloroquina, hidroxicloroquia, amiodarona etc.). É indutor da glicoproteína-P ABCB1, podendo elevar a concentração celular e o risco de toxicidade dos medicamentos substratos, o que possivelmente ocorre com sua associação à varfarina, digoxina, sinvastatina e morfina, por exemplo, que oferecem maior risco de reações adversas quando combinados com a azitromicina
Oseltamivir	Vômitos, cefaleia, náuseas, arritmias cardíacas, alterações neuropsiquiátricas, dermatites e hipersensibilidade	Pode aumentar o risco de sangramento se associado à varfarina
Lopinavir/ritonavir	Erupção cutânea, diarreia, vômitos, náuseas, hipertrigliceridemia, hipercolesterolemia, pancreatite, desconforto respiratório e prolongamento do intervalo QT	O ritonavir é um potente inibidor do citocromo P450 CYP3A4, a principal via de metabolização de diversos medicamentos, entre eles: eritromicina, haloperidol, dexametasona, prednisona, midazolam, fentanila, anlodipino, sinvastatina, ondansetrona, tramadol, atorvastatina, lopinavir e ziprasidona. Portanto, fármacos metabolizados por essa via quando associados a lopinavir/ritonavir podem sofrer redução no metabolismo e consequente acúmulo no organismo, ampliando o risco de toxicidade
Glicocorticosteroides	Reações dermatológicas, hiperglicemia, hipertensão, maior suscetibilidade a infecções, alterações gastrintestinais, neuropsiquiátricas e oftalmológicas	Associados aos AINES, podem provocar úlcera gástrica e hemorragia digestiva. São substratos da via CYP3A4 e podem ter seu metabolismo alterado por inibidores (claritromicina, azitromicina, itraconazol, voriconazol, lopinavir/ritonavir, atazanavir etc.) e indutores (carbamazepina, fenobarbital, fenitoína, rifampicina etc.) dessa via
Tocilizumabe	Hipercolesterolemia, hipertensão, dor abdominal, diarreia, cefaleia, rash, urticária, reações infusionais, maior suscetibilidade a infecções e insuficiência hepática	Pode reduzir a efetividade da imunização das vacinas. É indutor da via CYP3A4 e pode reduzir os níveis plasmáticos dos diversos medicamentos metabolizados por essa via
Heparina (de baixo peso molecular ou não fracionada)	Trombocitopenia, reações de hipersensibilidade, hemorragia e febre	Há maior risco de hemorragias quando associada a antiplaquetários e AINES. Seu efeito anticoagulante pode ser reduzido por derivados do estrógeno e da progestina

AINES - anti-inflamatórios não esteroides.

Fonte: adaptado de: DynaMed and Micromedex with Watson, UpToDate^®^, Lexicomp^®^ Drug Interactions, IBM Micromedex^®^ Drug Interections e ANVISA (bulário eletrônico).
